# Identification and characterization of PhoP regulon members in *Yersinia pestis *biovar *Microtus*

**DOI:** 10.1186/1471-2164-9-143

**Published:** 2008-03-27

**Authors:** Yingli Li, He Gao, Long Qin, Bei Li, Yanping Han, Zhaobiao Guo, Yajun Song, Junhui Zhai, Zongmin Du, Xiaoyi Wang, Dongsheng Zhou, Ruifu Yang

**Affiliations:** 1 State Key Laboratory of Pathogen and Biosecurity, Institute of Microbiology and Epidemiology, Beijing 100071, China

## Abstract

**Background:**

The transcription regulator PhoP has been shown to be important for *Y. pestis *survival in macrophages and under various *in vitro *stresses. However, the mechanism by which PhoP promotes bacterial intracellular survival is not fully understood. Our previous microarray analysis suggested that PhoP governed a wide set of cellular pathways in *Y. pestis*. A series of biochemical experiments were done herein to study members of the PhoP regulon of *Y. pestis *biovar *Microtus*.

**Results:**

By using gel mobility shift assay and quantitative RT-PCR, a total of 30 putative transcription units were characterized as direct PhoP targets. The primer extension assay was further used to determine the transcription start sites of 18 PhoP-dependent promoters and to localize the -10 and -35 elements. The DNase I footprinting was used to identify the PhoP-binding sites within 17 PhoP-dependent promoters, enabling the identification of PhoP box and matrix that both represented the conserved signals for PhoP recognition in *Y. pestis*. Data presented here providing a good basis for modeling PhoP-promoter DNA interactions that is crucial to the PhoP-mediated transcriptional regulation.

**Conclusion:**

The proven direct PhoP targets include nine genes encoding regulators and 21 genes or operons with functions of detoxification, protection against DNA damages, resistance to antimicrobial peptides, and adaptation to magnesium limitation. We can presume that PhoP is a global regulator that controls a complex regulatory cascade by a mechanism of not only directly controlling the expression of specific genes, but also indirectly regulating various cellular pathways by acting on a set of dedicated regulators. These results help us gain insights into the PhoP-dependent mechanisms by which *Y. pestis *survives the antibacterial strategies employed by host macrophages.

## Background

Plague, caused by *Yersinia pestis*, is one of the most dangerous, infectious diseases in the world [1]. *Y. pestis *has the ability of survival and growth within naïve macrophages both *in vivo *and *in vitro *[2,3]. It has been shown that *Y. pestis *is a facultative intracellular parasite that resides and grows within host macrophages during the early stages of infection [4]. Many virulence factors of *Y. pestis *have been tested for their ability to promote resistance to macrophage killing: pesticin, Pla, the pigmentation phenotype, and the plasmid pCD1-encoded type III secretion system, but they are all shown not to be required for bacterial survival in macrophages [2,5-7]. Besides the two-component regulatory system PhoP-PhoQ [8] and the *rip *operon [9], the determinants which allow the pathogen's survival and growth in the macrophages are still poorly understood.

A low Mg^2+ ^concentration is indicative of an intracellular environment as seen in macrophages, while a high Mg^2+ ^concentration denotes extracellular fluids [10]. PhoQ, an inner membrane-bound sensor-kinase, is inactive when bound with Mg^2+^; however, when the extracellular Mg^2+ ^concentration drops, Mg^2+ ^dissociates from PhoQ, leaving it activated. PhoQ then phosphorylates PhoP that either activates or represses transcription of specific target genes [11]. As a transcription factor, PhoP controls its target genes through binding to their promoter DNA regions. A 17 bp PhoP box sequence, which consists of a direct repeat of the hexanucleotide consensus (T/G)GTTTA, has been shown to be the conservative signals of the PhoP-binding sites in *Escherichia coli *[12,13]. This is essentially in agreement with the heptanucleotide consensus (T/G)GTTTA(A/T) sequence proposed in *Salmonella enterica *[14].

A *phoP *null mutant of *Y. pestis *shows a reduced ability to survive in macrophages and under *in vitro *conditions of low pH, oxidative stress, high osmolarity and antimicrobial peptides [8,15]. The mean lethal dose of this *phoP *mutant in mice is increased 75-fold in comparison with that of the wild-type strain [8]. In our previous work [16], cDNA microarray was used to determine the expression profile of a *Y. pestis phoP *null mutant grown under low Mg^2+ ^conditions, enabling the genome-wide screening for PhoP-dependent genes in *Y. pestis*.

In the present work, a total of 30 transcription units were characterized as the direct targets of PhoP. It was shown that PhoP was a global regulator governing a complex regulatory cascade in *Y. pestis*. The subsequent determination of transcription start sites, -10/-35 elements, and PhoP-binding sites enabled the mapping of PhoP-DNA interactions, and helped to model the PhoP-mediated transcriptional regulation. Various genes of protective or adaptive functions, identified to be under the direct and positive control of PhoP, might contribute to *Y. pestis*'s escape from the antibacterial strategies employed by host macrophages.

## Methods

### Bacterial strains

The wild-type (WT) strain 201 was isolated from *Microtus brandti *in the Inner Mongolia, China. It has an LD_50 _of less than 10 cells for mice by subcutaneous challenge. Strain 201 belongs to a newly established *Y. pestis *biovar *Microtus*, which is supposed to be avirulent to humans, although this biovar is highly lethal to mice [17]. The *phoP *null mutant of *Y. pestis *[16] was generated by using the one-step inactivation method based on the lambda phage recombination with the helper plasmid pKD46 [18].

### Bacterial growth and RNA isolation

Bacterial growth and RNA isolation were essentially the same as those described previously [16]. A chemically defined TMH medium [19] was used for cultivating the bacteria. Both the WT strain and the *phoP *null mutant were pre-cultivated at 26°C in the TMH medium containing 20 mM MgCl_2 _to an OD620 of about 0.6. Bacteria were harvested, washed twice with the TMH medium containing 10 μM MgCl_2_, and then 1:20 diluted into the fresh 10 μM Mg^2+ ^TMH medium. Both the strains were grown at 26°C until reaching the middle exponential growth phase (an OD620 of about 0.6) and then the cell cultures were transferred to 37°C for 1 h before harvest for RNA isolation.

Immediately before being harvested, bacterial cells were mixed with RNAprotect Bacteria Reagent (Qiagen) to minimize RNA degradation. Total RNA was isolated by using the TRIzol Reagent (Invitrogen). RNA quality was monitored by agarose gel electrophoresis and RNA quantity was measured by spectrophotometer.

### Preparation of His-PhoP

The entire coding region of the *phoP *gene of strain 201 was cloned into plasmid pET28a (Novagen). The recombinant plasmids encoding a His-PhoP fusion protein were transformed into *E. coli *BL21 (DE3) cells (Novagen). Expression of His-PhoP was induced by addition of 1 mM isopropyl-beta-D-thiogalactoside. The recombinant protein was purified under native conditions with a QIAexpressionist™ Ni-NTA affinity chromatography (Qiagen). All steps in the isolation procedure were performed at 4°C. The purified, eluted protein was concentrated to a final concentration of about 1.0 mg ml^-1 ^with the Amicon Ultra-15 (Millipore). The protein purity was verified by SDS-PAGE.

### Preparation of polyclonal antibody against His-PhoP

The six-week-old New Zealand white rabbits were immunized with the His-PhoP protein (100 μg/rabbit) emulsified with the Freund's complete adjuvant through the subcutaneous route, followed by the boost immunization every two weeks for another three times with the mixture of PhoP/Freund's incomplete adjuvant. The specific antibody in the serum was monitored by indirect ELISA. The blood was collected by the carotid bleeding under callisection and the serum was separated for IgG purification by the method of saturated ammonium sulfate. The purity of the antibody was verified by SDS-PAGE and its quantity was measured by UV spectrometry.

### Gel mobility shift assay (EMSA)

Primers (Additional file 1) were designed to amplify 400 to 500 bp region upstream of the start codon of each gene tested. EMSA was performed using the Gel Shift Assay Systems (Promega). The 5' ends of DNA were labeled using [γ-^32^P] ATP and T4 polynucleotide kinase. DNA binding was performed in a 10 μl reaction volume containing binding buffer [1 mM MgCl_2_, 0.5 mM EDTA, 0.5 mM DTT, 50 mM NaCl, 10 mM Tris-HCl (pH 7.5) and 0.05 mg/ml poly-(dI-dC)], labeled DNA (1000 to 2000 c.p.m/μl) and increasing amount of the His-PhoP protein. We included three controls in each EMSA experiment: i) specific DNA competitor (unlabeled promoter region of the same gene); ii) nonspecific DNA competitor [unlabeled promoter region of *purK*, one of the negative controls (see below)]; and iii) the supershift control (the antibody against His-PhoP was added for supershift by formation of DNA-PhoP-antibody complex). After incubation at room temperature for 30 min, the products were loaded onto a native 4% (w/v) polyacrylamide gel and electrophoresed in 0.5×TBE buffer for about 30 min at 220 V. Radioactive species were detected by autoradiography after exposure to Kodak film at -70°C.

### Real-time quantitative RT-PCR

Gene-specific primers (Additional file 1) were designed to produce a 150 to 200 bp amplicon for each gene. The contaminated DNA was removed from each 5 to 10 μg of total RNA by using the Ambion's DNA-free™ Kit. cDNA was generated by using 5 μg of total RNA and 3 μg of random hexamer primers. Real-time PCR was performed in triplicate using independent cultures and RNA preparations through the LightCycler system (Roche) together with the SYBR Green master mix, and with 1:25 dilution of cDNA as templates. To ensure that there was no contamination of genomic DNA, negative controls were performed by using 'cDNA' generated without reverse transcriptase as templates. Reactions containing primer pairs without template were also included as blank controls. 16S rRNA was used as an internal control for each RNA preparation. On the basis of the standard curves of 16S rRNA expression, the relative mRNA level was determined by calculating the threshold cycle (ΔCt) of each gene by the classic ΔCt method [20]. The RT-PCR data of 16S rRNA gene was used to normalize that of all the other genes. The transcriptional variation of the WT strain and the *phoP *null mutant for each gene was then calculated. A mean ratio of two was taken as the cutoff of statistical significance.

### Primer extension

An oligonucleotide primer (Additional file 1) was designed to be complementary to the RNA transcript of each gene from a suitable position (generally 80 to 150 bp downstream of the translation start site). Primer extension assay was performed using the Primer Extension System-AMV Reverse Transcriptase kit (Promega). The extension primers were end-labeled with [γ-^32^P] ATP. Three nanograms of each end-labeled primer were annealed with 10 to 30 μg of total RNA and extended according to the manufacturer's protocol. The same labeled primer was also used for sequencing with the *fmol*^® ^DNA Cycle Sequencing System (Promega). The products were concentrated and subjected to electrophoresis in a 6% polyacrylamide/8 M urea gel. The result was detected by autoradiography (Kodak film).

### DNase I footprinting

To obtain each promoter DNA fragment with a single ^32^P-labeled end, PCR amplification was performed with the promoter-specific primer pairs (see Additional file 1), in which either the sense or the antisense primer was end-labeled. The PCR products were purified by using MinElute reaction cleanup columns (Qiagen). Increasing amount of His-PhoP was incubated with the labeled DNA fragment (2 to 5 pmol) for 30 min at room temperature in a final volume of 10 μl containing binding buffer same as EMSA. Before DNA digestion, 10 μl of Ca^2+^/Mg^2+ ^solution (5 mM CaCl_2 _and 10 mM MgCl_2_) was added, followed by incubation for 1 min at room temperature. Then, the optimized RQ1 RNase-Free DNase I (Promega) was added to the reaction mixture, and the mixture was incubated at room temperature for 50 to 90s. The reaction was quenched by adding 9 μl of the stop solution (200 mM NaCl, 30 mM EDTA and 1% SDS) for 1 min at room temperature. The partially digested DNA samples were extracted with phenol/chloroform, precipitated with ethanol, and analyzed in a 6% polyacrylamide/8M urea gel. Protected regions were identified by comparison with the sequence ladders. For sequencing, the *fmol*^® ^DNA Cycle Sequencing System (Promega) was used. The results were detected as described above.

### Computational promoter analysis

The 500 bp region upstream of the start codon of each gene tested was retrieved with the *retrieve-seq *tool [21]. By using the *FUZZNUC *program [22], the *E. coli *PhoP box was matched within the PhoP sites as determined by DNase I footprinting. Homologues of the PhoP box identified from the PhoP sites were then collected and displayed by the *WebLogo *program [23] to generate a sequence logo; additionally, the position count matrixes were built from them by using the *consensus-matrix *and *convert-matrix *tools [21].

## Results

### Direct targets of PhoP

The comparison of mRNA profile of the WT *Y. pestis *with that of the *phoP *null mutant under the Mg^2+^-limiting condition [16] identified an array of 706 PhoP-dependent genes. This analysis had the difficulty in distinguishing between direct targets and indirect ones of PhoP. In the present study, the following genes (87 genes in total) were picked out from the above 706 ones for EMSA: i) those responsible for stress-adaptive or regulatory function; and ii) those with a homologue of *E*. *coli *PhoP box sequence in their promoter-proximal regions (with a cutoff of 2 bp mismatch). The EMSA was conducted to test the binding of His-PhoP to the 400 to 500 bp region upstream of the translation start site for each gene. Accordingly, a total of 30 transcription units gave the stable, positive results of EMSA (Table 2 and Additional file 3). To ensure the specificity of EMSA, two additional genes (*purK *and *atpG*) were included as negative controls. For selection of them as negative controls, the *patser-matrix *tool [21] was employed to match the PhoP matrix (see below) within their promoter regions, and both of them gave a very low value of weight score (a greater number of this score corresponds to a higher probability of presence of a PhoP-binding site).

^1 ^The gene IDs were derived from the genome annotation of *Y. pestis *CO92. Putative transcription units (30 in total) were boxed, and the vertical arrows indicated the transcriptional organization.

^2 ^The mRNA expression in the *phoP *null mutant was compared with that in the WT strain grown under low Mg^2+ ^condition.

^3 ^The data were present as the mean change of mRNA level for each gene under the paired growth conditions. The positive number stood for fold increased, while minus decreased.

^4 ^There was the discrepancy between the data determined by RT-PCR and microarray (a total of three genes); the subsequent primer extension assay verified the rationality of the RT-PCR results.

^5'^ND' indicated ^'^not done'.

The microarray analysis was able to semi-quantitatively identify genes under either positive or negative control by PhoP. However, microarray results are influenced by various factors, and thereby should be validated by at least one traditional method [24]. Accordingly, the real-time quantitative RT-PCR, using the same RNA preparations as the microarray analysis, was performed to validate the microarray data in Additional file 2. Except for three genes (*metJ*, *fruR *and *astC*), RT-PCR and microarray data showed a good agreement. Since RT-PCR and microarray gave the contradictory results for the above three genes, the further primer extension assay (Additional file 4) was conducted by using the same RNA preparations, which showed the creditability of the RT-PCR data (Additional file 2 and Additional file 5). Thereby, the positive or negative regulation of each selected gene by the PhoP was elucidated as shown in Additional file 2.

Taken together, we identified a total of 30 transcription units whose transcription was PhoP-dependent in response to Mg^2+ ^limitation, and these units should be the direct PhoP targets.

### Transcription start sites of direct PhoP targets

In addition to the above three genes (*metJ*, *fruR *and *astC*), we still performed the primer extension assay on another 15 genes (Additional file 4). These genes encoded either stress-adaptive or regulatory functions. A single primer extension product was detected for 12 of them. Two or more primer extension products were detected for *katA*, *uspB, pmrE, oppA*,*phoP *and *slyA*. Since the shorter extension products might represent the premature stops due to difficulties of polymerase in passing difficult sequences, only the longest product was chosen for the identification of each gene's transcription start site.

Accordingly, a transcription start site was identified for each of the above 18 genes, indicating a PhoP-dependent promoter was transcribed for each of them. The nucleotide number of each transcription start site was taken as '+1', and accordingly the promoter -10 and -35 elements for RNA polymerase(RNAP) recognition were predicted (see below).

### The PhoP-binding sites

To precisely determine the PhoP-binding sites of target genes, DNase I footprinting assay was performed on 17 PhoP-dependent promoter DNA regions (both coding and noncoding strands) (Additional file 6). DNase I footprinting results confirmed the direct binding of His-PhoP to these promoter regions *in vitro*. For each DNA fragment tested, His-PhoP protected at least one distinct region against DNase I digestion in a dose-dependent pattern. The size of footprint ranged from 25 to 62 bases with an average of 35 bases. These footprint regions were considered as the PhoP-binding sites. Interestingly, the *phoP *promoter region had two separated PhoP binding sites, while *sodB *had three (Additional file 6). Additionally, the *uspA *and *uspB *genes shared a single PhoP binding site (see below). In total, 19 PhoP-binding sites were detected for the 17 PhoP-regulated genes analyzed.

The His tag fused in the recombinant PhoP protein is positively charged, while DNA probe in the footprinting experiments is negatively charged. One may argue that there would be the possibility of nonspecific binding between His tag and DNA probe. Accordingly, the PCR-generated upstream DNA fragments of two genes *sodC *and *oppA *were employed as negative controls (Additional file 7). The DNA probes used here did not harbor the predicted PhoP box, compared with the corresponding ones in Additional file 6. No His-PhoP protected region was detected for the DNA probes tested, indicating the specificity of DNase I footprinting experiments.

### The PhoP regulatory motif in *Y. pestis*

**Figure 1:**
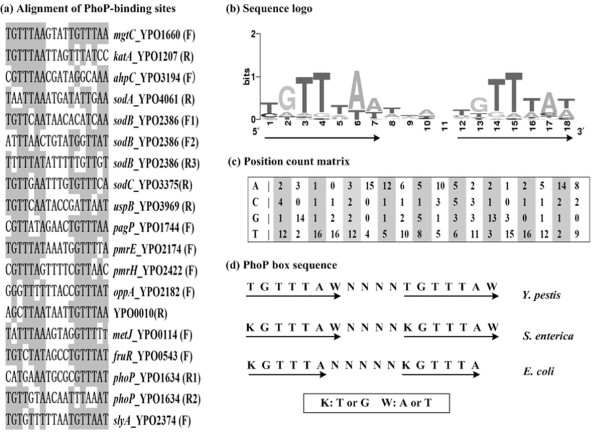
**Alignment of PhoP regulatory motifs in *Y. pestis***. (a) Homologues of the PhoP box, identified from the 19 PhoP binding sites determined by DNase I footprinting, were aligned and the conserved positions were highlighted. 'F' and 'R' in the brackets indicated the coding and noncoding stand, respectively. '1', '2' and '3' represented the numbers of PhoP-binding sites in a specific PhoP-dependent promoter (see Additional file 6 for details). (b) A sequence-Logo representation of the aligned direct repeat of the short consensus sequence (TGTTTAW). (c) Position count matrix describing the sequence logo. In the matrix, each row represents a position and each column a nucleotide. (d) An 18 bp PhoP box sequence (TGTTTAWN_4_TGTTTAW) of *Y. pestis *was generated by the sequence logo. The known corresponding ones for *E. coli *and *S. typhimurium *were shown as well.

Matching of the *E. coli *PhoP box within the above 19 PhoP binding sites (both coding and noncoding strands) revealed the tandem direct repeat in each of them. Then, an 18 bp footprint sequence covering each detecting regulatory motif was picked out for the alignment as shown in Figure 1(a). A sequence logo of the aligned direct repeats was given in Figure 1(b). In addition, position count matrix was built as shown in Figure 1(c), so as to statistically describe the alignment of the direct repeat elements. Finally, an 18 bp PhoP box sequence (TGTTTAWN_4_TGTTTAW) in Figure 1(d) was deduced from the sequence logo, which was very similar to those of *E. coli *and *S. enterica*.

### Organization of PhoP-dependent promoters

**Figure 2:**
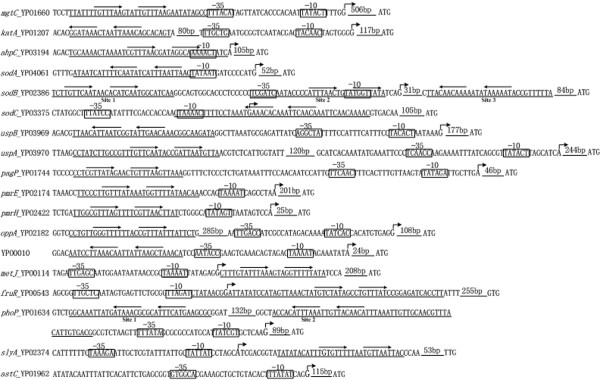
**Organization of PhoP-dependent promoters**. The DNA sequences derived from the genomic data of *Y. pestis *CO92 and the start codon of each gene was shown at the 3' terminal. The bent arrows indicated the transcription start sites. The nucleotide numbers were shown by taking the transcription start site as "+1". The predicted promoter -10 and/or -35 elements were boxed. The DNase I footprint regions (PhoP-binding sites) were underlined. The short consensus sites (TGTTTAW) were shown with arrows above.

As described above, we mapped PhoP-binding sites and transcription start sites within a collection of 17 PhoP-dependent promoters. These data enabled us to depict the organization of PhoP-dependent promoters (Figure 2) which gave a map of PhoP-DNA interaction within each promoter. For each target gene, translation/transcription start sites, promoter -10 and -35 elements, PhoP-binding sites, and PhoP box sequence were shown together.

**Figure 3:**
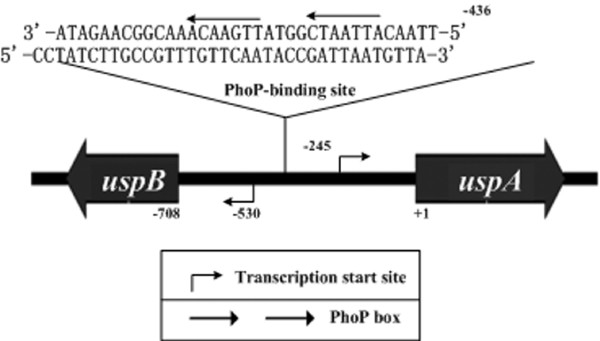
**Control of the *uspA *and *uspB *genes by PhoP**. The PhoP binding sites of the *uspA *and *uspB *genes were shown. The letters with arrows above indicated the PhoP box. "+1" indicated the first base pair within the coding region of *uspA*, while the upstream base pairs were shown with the minus numbers. The transcription start sites were displayed as well.

The products of *uspA *and *uspB *belong to the UspA family proteins with roles in defense against DNA damage. These two adjacent genes are transcribed with opposite direction in *Y. pestis *[25] and activated by PhoP (Additional file 2). DNase I footprinting identified a single PhoP-protected region for both coding and noncoding strands of their intergenic region (36 and 35 bp, respectively) (Figure 3). These two footprint sequences overlapped 34 bp, and a sequence resembling the PhoP box was found within this region. Thus, these two genes share a single PhoP-binding site to mediate their transcriptional activation in low Mg^2+ ^milieu.

## Discussion

### PhoP-dependent genes and direct PhoP targets

Experiments of *lacZ *reporter fusion in *S. typhimurium *[26,27] and *E. coli *[28] have shown that the PhoP regulatory protein affects more than 40 different genes, termed PhoP-activated and PhoP-repressed genes. By comparison of gene expression profiles between a wild-type strain and the isogenic mutant of *phoP *grown under low Mg^2+ ^conditions, cDNA microarray enables the genome-wide identification of PhoP-dependent genes in both *E. coli *[12,29] and *S. typhimurium *[30]. The *lacZ *fusion experiments can demonstrate whether the promoter activity of an upstream DNA fragment is dependent on PhoP, while microarray analysis can screen for genes whose transcription is affected by the mutation of *phoP *gene. Both of them cannot answer whether a gene is a direct or indirect target of PhoP.

Protein-DNA binding experiments, including EMSA, DNase I footprinting and chromatin immunoprecipitation, can be used to detect the direct target genes of PhoP. Accordingly, at least 22 transcription units have been proven to be the direct PhoP targets in *S. typhimurium *and/or *E. coli *(summarized in Additional file 8). In the present work, we identify a total of 30 transcription units under the direct control of PhoP in *Y. pestis *by using EMSA, DNase I footprinting, real-time RT-PCR and primer extension. Only three analogues (*slyA *[31], *phoP *[12,14] and *mgtCB *[14]) of them have been previously reported in *S. typhimurium *and/or *E. coli*. In addition, DNase I footprinting has demonstrated the binding of the *Salmonella *PhoP protein to the upstream DNA fragments of the *Y. pestis pmrF *and *pmrE *operons [32], which was confirmed in this study by using the *Y. pestis *PhoP protein. Thus, the present study disclosed 25 'novel' direct targets of PhoP, which encoded a wide range of functions including oxidative defense, universal stress response, synthesis and modification of LPS, oligopeptide transport, peptidoglycan remodeling, regulators, and various/unknown functions (Additional file 2).

### The Mg^2+^-responsive PhoP is a global transcriptional regulator in *Y. pestis*

Genomic transcriptional profiling of the *phoP *null mutant of *Y. pestis *in response to low Mg^2+ ^stimulus revealed at least 706 PhoP-dependent genes, representing approximately 16% of the total protein-coding capacity of the *Y. pestis *genome [16]. These genes are distributed among almost all the functional classes according to the genome annotation of *Y. pestis *CO92 (see Additional file 9). In our present work, the detected 30 direct PhoP targets including nine genes (YPO0010, YPO0414, YPO0736, YPO1279,*metJ*, *fruR*, *lacI*, *phoP *and *slyA*) that encode broad regulatory functions (Additional file 2).

The general features of *Y. pestis *PhoP can be summarized as follows: i) it regulates a large number of genes that belong to a wide set of functional classes; ii) it not only directly controls the expression of specific genes, but also indirectly regulates various cellular pathways by acting on a set of local regulators; and iii) it functions as either an activator or a repressor depending on the target promoters. A conclusion could be postulated that the Mg^2+^-responsive PhoP is a global transcriptional regulator that governs a complex of regulatory cascades in *Y. pestis*.

### PhoP-promoter DNA interactions for transcriptional regulation

As shown above, *Y. pestis *PhoP binds to DNA regions at various positions from the RNA polymerase-binding site (-10 and -35 elements), i.e., upstream, overlapping or downstream of the promoter -35 elements (Figure 2). Thus, the PhoP-dependent promoters identified herein could be grouped into the following three categories: i) Class I promoters, which can be found in the *mgtC, katA, sodB*(Site1), *uspB, uspA, pagP, oppA*, YPO0010 and *phoP *promoters, contain PhoP-binding sites upstream of the promoter -35 element. Class I PhoP-dependent promoters may have a class I regulation that depends on the RNA polymerase α subunit C-terminal domain (αCTD) for function [33]; ii) Class II promoters containing PhoP-binding sites overlapping the DNA binding site of RNAP are found in the *ahpC, sodA, sodB*(Site2),*pmrE *and *pmrH *promoters. There may be a class II regulation for these promoters, which requires the RNA polymerase σ subunit C-terminal domain (σCTD) or the α-subunit surface for function[34]; and iii) Class III promoters, downstream of the DNA binding site of RNAP, are seen in the *sodB*(Site3), *sodC, metJ, fruR *and *slyA *promoters. Although unusual, this kind of promoter have been found in *Salmonella*, where SlyA binds downstream of the transcription start sites of *ugtL *and *slyA *[31,35].

**Figure 4:**
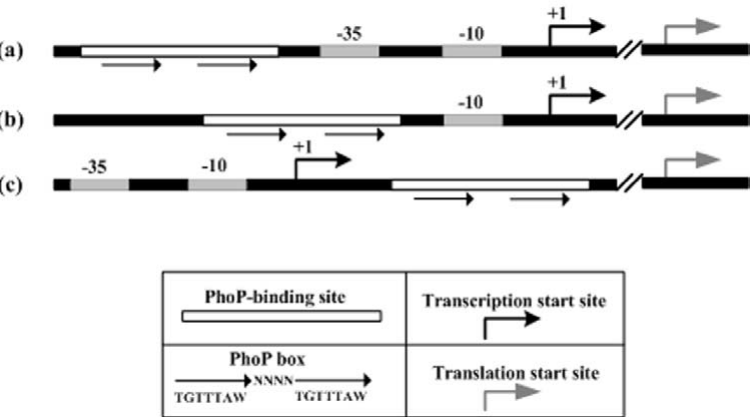
**Representation of PhoP-regulated promoters**. Three classes of PhoP-dependent promoter were shown. *Y. pestis *PhoP binds to DNA regions at various positions from the RNA polymerase-binding site (-10 and -35 elements), i.e., upstream (a), overlapping (b) or downstream (c) of the promoter -35 elements. The corresponding experimental promoters could be found in Figure 2.

Taken the above together, we proposed the modes for PhoP-promoter DNA interaction (Figure 4), which depicted the general rules of PhoP-promoter DNA recognition for initiating the transcriptional regulation. Similar classification of PhoP-dependent promoters has been proposed in *E. coli *and *S. enterica *[36].

Two PhoP-binding sites, site 1 and site 2, were identified within the *phoP *promoter (Additional file 6). Fifteen pmol of PhoP gave a complete protection on the site 1, while for the site 2, 25 pmol of PhoP still showed an incomplete protection (Additional file 6). It has been proposed that the doses of DNA-binding transcription factor required for the transcriptional activation of its target genes are related to their binding affinity [37]. Accordingly, the PhoP's affinity to site 1 was stronger than that to site 2. Both site 1 and site 2 are located upstream of the -35 element.

We defined the PhoP sites within a collection of 17 PhoP-dependent promoters in *Y. pestis*. A relatively larger collection of PhoP-binding sites here promoted us to perform the sequence alignment, leading to the identification of a PhoP box (very similar to those in *E. coli *and *S. enterica*) as well as the position count matrix (Figure 1). All of them represent the conserved signals for PhoP recognition in *Y. pestis*. The box is a contiguous oligonucleotide, and thus it can not show much of the information originally presented in these PhoP sites. Representation of consensus patterns with the position count matrix is able to give a full description of the uneven composition in each position, i.e., some nucleotides occurred much more frequently than others. Thus, the matrixes presented here will over-represent the PhoP-recognized DNA binding sites in *Y. pestis *more accurately than the classic 18 bp box sequence.

The determination of PhoP regulatory motifs in *Y. pestis *promoted us to identify additional genes potentially under the direct control of the PhoP. Briefly, the 500 bp fragment upstream of each PhoP-dependent gene determined by cDNA microarray was scanned with the position count matrix in Figure 1(c) by using the *patser-matrix *tool [21]. This analysis generated a weight score for each promoter DNA. A greater value of this score corresponds to a higher probability of presence of a PhoP-binding site. When seven was taken as the cutoff value, a total of 88 genes or possible operons, which could be assigned into nine functional categories, were picked out as the potential direct PhoP targets (see Additional file 10). Searching the upstream regions of genes for the PhoP consensus sequences can help us identify the candidate targets regulated directly by the PhoP. However, the mere presence of a PhoP consensus does not necessarily mean that the PhoP must bind and regulate the target. Either EMSA or DNase I footprinting is required to determine whether it is a functional target for PhoP-specific binding.

### PhoP-regulated functions likely contribute to intracellular growth of *Y. pestis*

*Y. pestis *is considered as a facultative intracellular pathogen that is capable of surviving and growing within macrophages during its early stage of infection. When *Y. pestis *cells are phagocytosed by a macrophage, they are usually found in a vacuole, the phagolysosome [38]. The phagolysosome has evolved an array of mechanisms to kill or inhibit the evading bacteria, including the activated oxygen species, an acidic pH as low as 4.0, the abundant antimicrobial peptides, and the limited availability of Mg^2+^.

Disruption of the *phoP *gene renders *Y. pestis *more sensitive to low pH, oxidative stress and antimicrobial peptides that are found to be killing mechanisms for invading pathogen by macrophage [8,15]. When the *phoP *gene of *Y. pestis *strain 201 used in this study was disrupted using a lambda Red recombination system [16], similar phenotypes of this *phoP *null mutant were also observed (data not shown).

Herein, a combined use of microarray, EMSA, RT-PCR, primer extension, and DNase I footprinting demonstrated that the PhoP could directly mediate the transcriptional activation of transcription units encoding functions that are protective against or adaptive to the killing mechanisms used by host macrophages: i) five genes (*sodA*, *sodB*, *sodC*,*katA*, and *ahpC*) encoding detoxification enzymes for scavenging oxidative stress; ii) two (*uspA *and *uspB*) playing roles in protection against DNA damage [39]; iii) three (*pmrHFIJKLM*, *pmrE *and *pagP*) for LPS modification enzymes that play key roles in the bacterial resistance to antimicrobial peptides; and iv) *mgtCB *encoding a major Mg^2+ ^transport system. In particular, the later two groups of genes has been shown to be regulated by the PhoP and required for the intra-macrophage survival of *S. typhimurium*, a classic facultative intracellular pathogen [11,40]. In addition, it has been reported that the *pmrE *and the *mgtC *function independently to promote early survival of *Y. pestis *in the macrophage phagosomes [41].

The above data lead us to an assumption that, in *Y. pestis*, the PhoP responds to the low Mg^2+ ^signal in host macrophages and mediates the transcriptional activation of various protective or adaptive functions. These functions, together, most likely make *Y. pestis *escape from macrophage-induced killing mechanisms. Further biochemical and phenotypic investigations on these candidate genes should be implemented to validate this hypothesis.

## Conclusion

A total of 30 transcription units (single-genes or putative operons) were characterized as direct PhoP targets in the present work, of which 25 appeared to be disclosed for the first time. In particular, 11 of them encoded various protective or adaptive functions (Mg^2+ ^transport, oxidative defense, universal stress response, and synthesis and modification of LPS), and they were positively controlled by PhoP, providing an array of candidate genes for future investigation on their contribution to the intracellular survival of *Y. pestis *in host macrophages. Additional nine of them encoded broad regulatory functions. Accordingly, the Mg^2+^-responsive PhoP could be proposed as a global regulator that governs a complex of regulatory cascade in *Y. pestis*.

Transcription start sites, promoter -10/-35 elements, and PhoP-binding sites were dissected within the upstream DNA regions of 17 PhoP-dependent genes or operons, providing a good basis for mapping PhoP-promoter DNA interactions and for modeling PhoP-mediated transcriptional regulation. Alignment of the detecting PhoP sites generated the PhoP regulatory motif of *Y. pestis*, in formats of both a continuous oligonucleotide (PhoP box) and the position count matrix, which would be useful for computational searching of additional, potential PhoP targets, even within the whole genome. Biovar *Microtus *strain 201 used in this study is highly lethal to mice, but avirulent to humans. Results obtained from this biovar should have their relevance for human-pathogenic strains. However, remodeling of regulatory cascades/networks likely contributes to the phenotypic divergences of difference biovars [17,42]. Further attempts should be conducted to investigate the differences or similarities between human-virulent and -avirulent *Y. pestis *isolates concerning PhoP-mediated regulation.

## Authors' contributions

DZ and RY conceived the study and designed the experiments. YL, HG, QL, BL, YH, ZG, YS, JZ, ZD, and XW performed the experiments. YL and DZ carried out the data mining including bioinformatics analyses. The manuscript was written by YL and DZ, and revised by RY. All authors read and approved the final manuscript.

## Supplementary Material

Additional file 1 Primers used in this study. Click here for file

Additional file 2 A collection of 30 PhoP regulon members in *Yersinia pestis*. Click here for file

Additional file 3 Electrophoretic mobility shift assays. Primers were designed to amplify the 400 to 500 bp DNA region upstream of the translation start site for each gene. Each upstream promoter DNA fragment was radioactively labeled, incubated with His-PhoP, and then subjected to native gel electrophoresis. The band of free promoter DNA disappeared with increasing amount of His-PhoP, and a retarded DNA band with decreased mobility turned up, which presumably represented the PhoP-DNA complex. With addition of the specific polyclonal antibody against His-PhoP, a "supershift" due to the formation of DNA-PhoP-antibody complex could be observed. A model graph of EMSA was shown as well. Click here for file

Additional file 4 Primer extension assays. Primer extension was performed by using RNA isolated from the exponential-phase of both the wild-type (WT) and *phoP *null mutant (*phoP*^-^) of *Y. pestis *grown in the chemically defined TMH medium with 10 μM of Mg^2+^. An oligonucleotide primer was designed to be complementary to the RNA transcript of each gene at a suitable position. The primer extension products were analyzed with a 6% acrylamide sequencing gel. Lanes C, T, A and G represented the Sanger sequencing reactions. The yield and length of each primer extension product can be used to map the 5' terminus of the RNA transcript, and thus the transcription start site. The transcription start sites were underlined. Click here for file

Additional file 5 Raw data for real-time PCR. Click here for file

Additional file 6 DNase I footprinting assays. The DNase I footprinting assay was performed on both the coding and noncoding strand of the promoter fragments generated by PCR. The radio-labeled promoter DNA was incubated with increasing amount of the His-PhoP (lanes 1, 2, 3, 4, and 5 contained 0, 5, 15, 25 and 35 pmol, respectively). After the partial digestion with DNase I, the resulting fragments are analyzed by denaturing gel electrophoresis. Lanes G, A, T and C represented the Sanger sequence reactions. On the right-hand side, the PhoP protected regions (bold line) were indicated, and the corresponding DNA sequences of footprints were shown from the bottom (5') to the top (3'). The overlapping of footprints on both strands of each gene was shown at the bottom. Click here for file

Additional file 7 Negative controls for DNase I footprinting assays. See Additional file 6 for the technical notes. The His tag fused in the recombinant PhoP protein is positively charged, while DNA probe in the footprinting experiments is negatively charged. One may argue that there would be the possibility of specific interaction between His tag and DNA probe. Accordingly, the PCR-generated upstream DNA fragments of two genes *sodC *and *oppA *were employed as negative controls. The DNA probes used here did not harbor the predicted PhoP box, compared with the corresponding ones in Additional file 6. No His-PhoP protected footprint region was detected for non-coding or coding strand of *sodC *and *oppA*, indicating the specificity of DNase I footprinting experiments. Click here for file

Additional file 8 Direct PhoP targets in *Escherichia coli *and *Salmonella typhimurium*. Click here for file

Additional file 9 Distribution of PhoP-dependent genes in *Yersinia pestis *as revealed by microarray. Click here for file

Additional file 10 Predicted direct PhoP target genes in *Yersinia pestis *by matrix matching. Click here for file
