# Supplementary material for: Identification and characterization of PhoP regulon members in Yersinia pestis biovar Microtus
Source: BMC Genomics. 2008 Mar 27;9:143. doi: 10.1186/1471-2164-9-143 (PMC2322996; doi:10.1186/1471-2164-9-143)
Supplement: Additional file 1 — Primers used in this study. [file 1471-2164-9-143-S1.doc]

**Supplementary Table S1. Primers used in this study**

| Gene ID | EMSA (Sense/antisense)(5’→3’) | RT-PCR (Sense/antisense)(5’→3’) | DNase I footprinting (Sense/antisense)(5’→3’) | primer extension(5’→3’) |
| --- | --- | --- | --- | --- |
| CO92-YPO1660 | CGTGATGTTATGTCGTTATCG/GATATACCGAGAGGGTGCTG | ACAATGGCGTCAGCGTATG/CACCCGCACCAAGGAAAC | CGTGATGTTATGTCGTTATCG/GCAAGCAACAAATCAATAGAG | GCAAGCAACAAATCAATAGAG  ACTTTATTTGGGGATGCACAG  GATATACCGAGAGGGTGCTG |
| CO92-YPO1207 | CGCCCGTGTTTGTCCC/GCGGTTTTGGGTTTAAGC | TGTCCGCTTCTCCACTGTG/GCAGGTTGGTACGAGGGTC | GCACACGCCACTAAATG/GCTATTGCTGTGGGAACTGAC | AATCCTTTCTTCTTGCTCAT |
| CO92-YPO3194 | GTGGTCAGAGCGTTGG/AACTATCGTAACAGCATAGG | GCAGACCAGCCGTCCTATTC/CTTCGCCAATACCGCCTTTATC | ATGTTCTTTGCTGTCTACCC/GACGAGTTACCAGAACCATA | AACTATCGTAACAGCATAGG  GACGAGTTACCAGAACCATA |
| CO92-YPO4061 | TGATCGACAATCCACACC/CATAGTGTAGGACGGTGC | GCCACGCTAACCACAGTC/CCAGCCAGAACCGAAACG | GCGTAAAACGTCAAAAAGAAGCG/AGGCATCATAAGCATAAGGCA | AGGCATCATAAGCATAAGGCA |
| CO92-YPO2386 | TTCAGTCTCCTCGTACCG/GTGCTCAGGGTGTTTGC | ATTGCCTGTCGCCAAATGG/CAATCGCCAATGTCCCATCC | ACCTCTAGCCCTAACTGAC/GTGCTCAGGGTGTTTGC | GTGCTCAGGGTGTTTGC |
| CO92-YPO3375 | CTATGAGCCGTTCTGACC/CTTCATACTCCCGCACAC | GCACCGCCACCTATCCC/TGACTCCACATGCCATCCG | TCAGATGCTGGCTTGCTTAG/GTCTGGTTTAACCTTGTC  CGGTCTGAAAGAAGTTAAAGGC/CTAAGCAAGCCAGCATCTGA(negative control) | GTCTGGTTTAACCTTGTC |
| CO92-YPO3969 | TGCATCTTGTCGGACCG/CGGCTCCTATCGTTCGC | TCTCTGCGTGCTCTGCTAG/CGGCGGATAAATTCGGGATC | GCAAGGTTAATGTTTGAGTAAGG/CGGCTCCTATCGTTCGC | CGTTCGCTTTCCTGTAATGG |
| CO92-YPO3970 | GCCGTTTGTTCAATACCG/CTCAACCTACCGAATGGG | TGACGTTAATCTCGGCGATATG/ATCCTGATGATGACCACACAAG | CAATACCCTATCTGAACG/GAATAGCGAGGATTTGAT | CCAATAAGTCACTAAACTCTACCGC |
| CO92-YPO1744 | ACCGCCAAAAACATCACG/ATCTCTAATGCAGATGAGGG | GAACCGATTCTGGGCTATGG/GGTGGTTTGTAGGGAGAGTC | TCACTCAACAACGGGAAAGC/ATCTCTAATGCAGATGAGGG | GAAACACACCGCTAAGAATGC |
| CO92-YPO2174 | GCTTAACAATGGTGTCCC/ACTCCCAGTGATTATCGG | ATTTGCTGTCTGGGGATTGG/CCATAAATGCGTTGCGTCTC | GAAGCATGGCAGTGTCAG/ACTCCCAGTGATTATCGG | ACTCCCAGTGATTATCGG |
| CO92-YPO2422 | GGTTGCTGGCTCAAGGG/GAGCGTCACCTGCTATGG | ATATCCACGCCGCTATTGC/CTTCATCAACACGCACCATG | CTTCTCCTATTCTCATCA/CTTCACTACCTATTGCTGGC | CTTCACTACCTATTGCTGGC |
| CO92-YPO2182 | TCTTTCGGTCCCTGTTGG/AATTTGCGTTACCTGGGG | AGGCGTACAACTGGCTGAG/CAACTCTCTGCGGCTCCC | TCTATGAGGCTGTTAGGAATC/CGAGTAAATGGCAGCAATA  ATTGCTGCCATTTACTCG/TTCTTCTTTGTGATGTTGGT(negative control) | TTCTTCTTTGTGATGTTGGT |
| CO92-YPO1715 | TTCTTTCCCGACACTACG/ATCGCGGTTATCATGACC | AACAACCTCAACGACACCTC/GCCACAACATCAGACGGAG |  |  |
| CO92-YPO0010 | AATAGGCATCTTGACCGAAG/CTGTTTCACTTCGGTATTGG | CTGGCAGCGAAGGGGATG/GGCTTGTGGTTCCAACGATG | GGAGTTCCTGACAGCCTGC/GCATGGTGATTTGTCCTATCAGTC | CCCGCTTCATATTCTCCTGC |
| CO92-YPO0114 | ACTGGAAAGGTGAATCGG/GCCCGATTTTCACTGTTC | GTGGAACGGCGAGTATGTC/TTCACTGTTGGTAGCATGGC | ATGTTACGCCAGAGTAGACGCAC/CATCACCGAAATACCCTTAG | GCCCGATTTTCACTGTTC |
| CO92-YPO0414 | GGCATGGACCTGACCG/TATTGCCGCTTTCTTGCC | GCGTTGGTGCCAGATATGC/CCGTCACCTCATCCGTCAC |  |  |
| CO92-YPO0543 | GCGGCTGGTAACAGAGC/GCCGAGAGCCAGCATCC | CAGTATTGGCAGTGGCACAG/TGCGGCTTAGGCTTAGAGTG | GCGGCTGGTAACAGAGC/CAAGCGAACGATCCTGATGAAC  GCGGCTGGTAACAGAGC/CTCCAGAGCGATCTAACCTC | CAAGCGAACGATCCTGATGAAC |
| CO92-YPO0736 | ACGAAGACGACACAATGC/AAAATGCTTGCCATGATTTG | GCGGAGACTAACGAAGAACC/GCACCAATAACGCAATAGCC |  |  |
| CO92-YPO0849 | TCTCGCCATAGGTATCGG/ACCCAATCACCCGAATCC | GGGAGAGAAGTCGGTCATTG/AGTTTGGCAGGCAGTATCG |  |  |
| CO92-YPO1279 | CGCCCATTGTACTGCTTG/AAACCGTGATGCCCAAAC | TCTACGCCAATGTCTGATGC/CTTCACGCACCACACTACG |  |  |
| CO92-YPO1634 | TGATGCCAGCAAAGACG/CGCTCATTATGTAGGTGC | ATCTGGAAGAGGTCATTGC/CTGCGTTGCGGATAAGG | TGATGCCAGCAAAGACG/GCATCGCTTGGTCACTGACT  GGCGAGTCAGTGACCAAGCG/CGCTCATTATGTAGGTGC | ACCCGCATACACCAATCCTT |
| CO92-YPO2374 | ATCCATCTGGCTATGTCG/TATACCGTCGATGCTAGG | TTACCACCAGAGCAATCACAG/ATCACGCCATCAACCTGTTC | ACCAAATCTGAAAGCGAGGCG/GTGCTAGATCAGATCCTAATGTCG | GTGCTAGATCAGATCCTAATGTCG |
| CO92-YPO0017 | GAGGGGGCTTTTGTTGC/TCTGACGCTAAGAATGCC | TTACAGCAAGAGCCAGAACC/CGCCAACACCGACATCC |  |  |
| CO92-YPO0498 | ATGCGGGCGGGAAAGG/ACGTCATCGTGCTAATGG | ACAAGGCTTCTCCCACCG/AGAGGAAGTTCAGGTAGCCG |  |  |
| CO92-YPO0860 | TTGACCCATCCCGTAAGC/GCTCCTGGTGCGTTCAAC | AGAACATCCTGCGGTAACAC/GCCATCGTGACCACATCTAC |  |  |
| CO92-YPO1937 | TCTTATTCCATGGGTTAGATAC/TTCTTGTCTGTCGGCAATC | AGCGTGATATGGCGTATAGG/AGAGTGCGGCGGATAGC |  |  |
| CO92-YPO1962 | AATGCACTTGATTGAGGC/TTGCCCCCTTTAATCAGC | GCCGCACGCTGTTTACG/CGCCCTCACCCTGAATGG |  | TTGCCCCCTTTAATCAGC |
| CO92-YPO2168 | AGGGAAACTAGTCGCATC/GGTCAGCACCGTTTTTGG | ACGAGAGTGGCTGGAACG/AGCCGCCAGTGGTTGAC |  |  |
| CO92-YPO3766 | AGAGTGAATCAGCAACGC/TAAGCGCATGTGATCTGG | AGCGAAGACAGCCTTGATTG/TCCTCCACCTAGTGCGTATC |  |  |
| CO92-YPO4116 | AAGGTGATGATGTTTGGC/GCCGCAGGTGTATTAGC | TTCTGTTGGGCGGCATTATC/CAGGCACGGCAATAATCAGG |  |  |
| CO92-YPO3077*1 | CACCACGCTCAATGTTCC/CTCGGATCAGGGTTGTCC |  |  |  |
| CO92-YPO4122*2 | TCTCCGTATCCCGTGTGG/AAGCAGGACCGCATAGC |  |  |  |
| * negative control: predicted score for *1 is 0.82,*2 is 0.71 | |  |  |  |
