# Supplementary material for: Identification and characterization of PhoP regulon members in Yersinia pestis biovar Microtus
Source: BMC Genomics. 2008 Mar 27;9:143. doi: 10.1186/1471-2164-9-143 (PMC2322996; doi:10.1186/1471-2164-9-143)
Supplement: Additional file 2 — A collection of 30 PhoP regulon members in Yersinia pestis. [file 1471-2164-9-143-S2.doc]

## Supplementary Table S1. A collection of 30 PhoP regulon members in Yersinia pestis

| **Microarray**^2, 3^ | **RT-PCR**^2, 3^ | **Primer extension**^2^ | **PhoP regulation** | **EMSA** | **Gene ID**^1^ | **Gene name** | **Product** |
| --- | --- | --- | --- | --- | --- | --- | --- |
| **Mg^2+^transport system** | | | | | | | |
| -4.90 | -11.16 | PhoP-activated | Activated | + | YPO1660  | *mgtC* | Mg(2+) transport ATPase protein C |
| -12.36 | -4.22 |  |  |  | YPO1661 | *mgtB* | Mg(2+) transport ATPase protein B |
| **Oxidative defense** | | | | | | | |
| -8.80 | -6.44 | PhoP-activated | Activated | + | YPO1207 | *katA* | Catalase |
| -5.06 | -2.86 | PhoP-activated | Activated | + | YPO3194 | *ahpC* | Alkyl hydroperoxide peroxidase subunit C |
| -4.81 | -17.53 | PhoP-activated | Activated | + | YPO4061 | *sodA* | Superoxide dismutase [Mn] |
| -3.22 | -14.34 | PhoP-activated | Activated | + | YPO2386 | *sodB* | Superoxide dismutase [Fe] |
| -3.72 | -4.92 | PhoP-activated | Activated | + | YPO3375 | *sodC* | Superoxide dismutase [Cu-Zn] precursor |
| **Universal stress responsive genes** | | | | | | | |
| -3.04 | -3.72 | PhoP-activated | Activated | + | YPO3969 | *uspB* | Universal stress protein B |
| -11.23 | -4.16 | PhoP-activated | Activated | + | YPO3970 | *uspA* | Universal stress protein A |
| **Oligopeptide transport system** | | | | | | | |
| -2.27 | -7.00 | PhoP-activated | Activated | + | YPO2182 | *oppA* | Periplasmic oligopeptide-binding protein precursor |
| -2.18 |  |  |  |  | YPO2183 | *oppB* | Oligopeptide transport system permease protein |
| -3.47 |  |  |  |  | YPO2184 | *oppC* | Oligopeptide transport system permease protein |
| -5.46 |  |  |  |  | YPO2185 | *oppD* | Oligopeptide transport ATP-binding protein |
| -3.52 |  |  |  |  | YPO2186 | *oppF* | Oligopeptide transport ATP-binding protein |
| **Peptidoglycan remodeling** | | | | | | | |
| -4.97 | -5.13 |  | Activated | + | YPO1715 | *ybjR* | Probable N-acetylmuramoyl-L-alanine amidase |
| **Synthesis and modification of LPS** | | | | | | | |
| -4.52 | ND^5^ | PhoP-activated | Activated | + | YPO1744 | *pagP* | Putative lipid A palmitoyltransferase |
| -15.78 | -4.15 | PhoP-activated | Activated | + | YPO2174 | *ugd/pmrE* | Putative nucleotide sugar dehydrogenase |
| ND^5^ |  |  |  |  | YPO2416 | *pmrM* | putative membrane protein |
| ND^5^ |  |  |  |  | YPO2417 | *pmrL* | putative membrane protein |
| -2.52 |  |  |  |  | YPO2418 | *pmrK* | Undecaprenyl phosphate-alpha-L-Ara4N transferase |
| -3.34 |  |  |  |  | YPO2419 | *pmrJ* | Conserved hypothetical protein |
| -9.58 |  |  |  |  | YPO2420 | *pmrI* | UDP-L-Ara4N formyltransferase |
| -15.60 |  |  |  |  | YPO2421 | *pmrF* | Undecaprenyl-phosphate Ara4FN transferase |
| -12.36 | -25.00 | PhoP-activated | Activated | + | YPO2422 | *pmrH* | UDP-Ara4O aminotransferase |
| **Regulators** | | | | | | | |
| -2.20 | -4.37 | PhoP-activated | Activated | + | YPO0010 |  | Putative GntR-famly transcriptional regulator |
| 2.22 | -11.83 | PhoP-activated^4^ | Activated | + | YPO0114 | *metJ* | Transcriptional repressor protein |
| -3.93 | -5.35 |  | Activated | + | YPO0414 |  | Putative transcriptional regulator |
| 2.11 | -3.14 | PhoP-activated^4^ | Activated | + | YPO0543 | *fruR* | Putative fructose repressor |
| -5.06 | -3.13 |  | Activated | + | YPO0736 |  | Putative regulatory protein |
| -2.64 | -2.62 |  | Activated | + | YPO0849 | *lacI* | Lactose operon repressor |
| -4.25 | -6.27 |  | Activated | + | YPO1279 |  | Putative transcriptional regulatory protein |
|  | -292.58 | PhoP-activated | Activated | + | YPO1634 | *phoP* | Response regulator protein |
| 8.84 | 5.51 | PhoP-repressed | Repressed | + | YPO2374 | *slyA* | MarR-family transcriptional regulatory protein |
| **Various/unknown functions** | | | | | | | |
| -3.10 | -4.15 |  | Activated | + | YPO0017 | *polA* | DNA polymerase I |
| -2.75 | -15.18 |  | Activated | + | YPO0498 |  | Hypothetical protein |
| -2.16 | -2.23 |  | Activated | + | YPO0860 |  | Sugar-binding periplasmic protein |
| 2.82 | 7.20 |  | Repressed | + | YPO1937 | *ansP* | L-asparagine permease |
| 4.61 | -9.03 | PhoP-activated^4^ | Activated | + | YPO1962 | *astC* | Succinylornithine aminotransferase |
| -2.46 | -5.32 |  | Activated | + | YPO2168 | *xthA* | Exodeoxyribonuclease III |
| -4.84 | -3.15 |  | Activated | + | YPO3766 | *fadB* | Fatty acid oxidation complex alpha subunit |
| -2.08 | -6.46 |  | Activated | + | YPO4116 | *pstC* | Putative phosphate transport system permease |

^1^ The gene IDs were derived from the genome annotation of *Y. pestis* CO92. The vertical arrows indicated the transcriptional organization of the putative transcription units.

^2^ The mRNA expression in the *phoP* null mutant was compared with that in the WT strain grown under low Mg^2+^ condition.

^3^ The data were present as the mean change of mRNA level for each gene under the paired growth conditions. The positive number stood for fold increased, while minus decreased.

^4^ There was the discrepancy between the data determined by RT-PCR and microarray (a total of three genes); the subsequent primer extension assay verified the rationality of the RT-PCR results.

^5 ‘^ND’ indicated ‘not done’.
