# Supplementary figures and images for: Identification and characterization of PhoP regulon members in Yersinia pestis biovar Microtus
Source: BMC Genomics. 2008 Mar 27;9:143. doi: 10.1186/1471-2164-9-143 (PMC2322996; doi:10.1186/1471-2164-9-143)

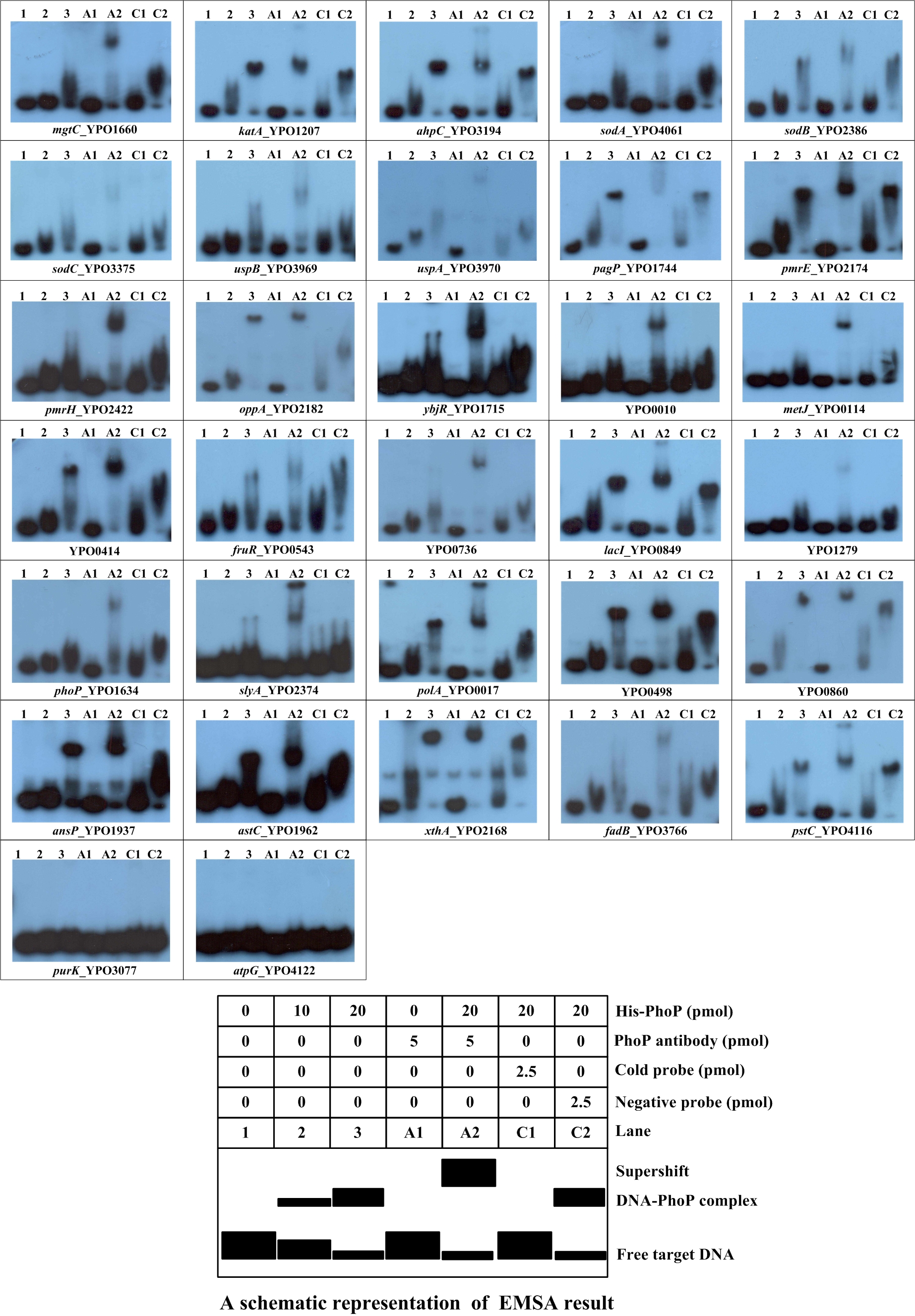

Supplement: Additional file 3 — Electrophoretic mobility shift assays. Primers were designed to amplify the 400 to 500 bp DNA region upstream of the translation start site for each gene. Each upstream promoter DNA fragment was radioactively labeled, incubated with His-PhoP, and then subjected to native gel electrophoresis. The band of free promoter DNA disappeared with increasing amount of His-PhoP, and a retarded DNA band with decreased mobility turned up, which presumably represented the PhoP-DNA complex. With addition of the specific polyclonal antibody against His-PhoP, a "supershift" due to the formation of DNA-PhoP-antibody complex could be observed. A model graph of EMSA was shown as well. [file 1471-2164-9-143-S3.doc]

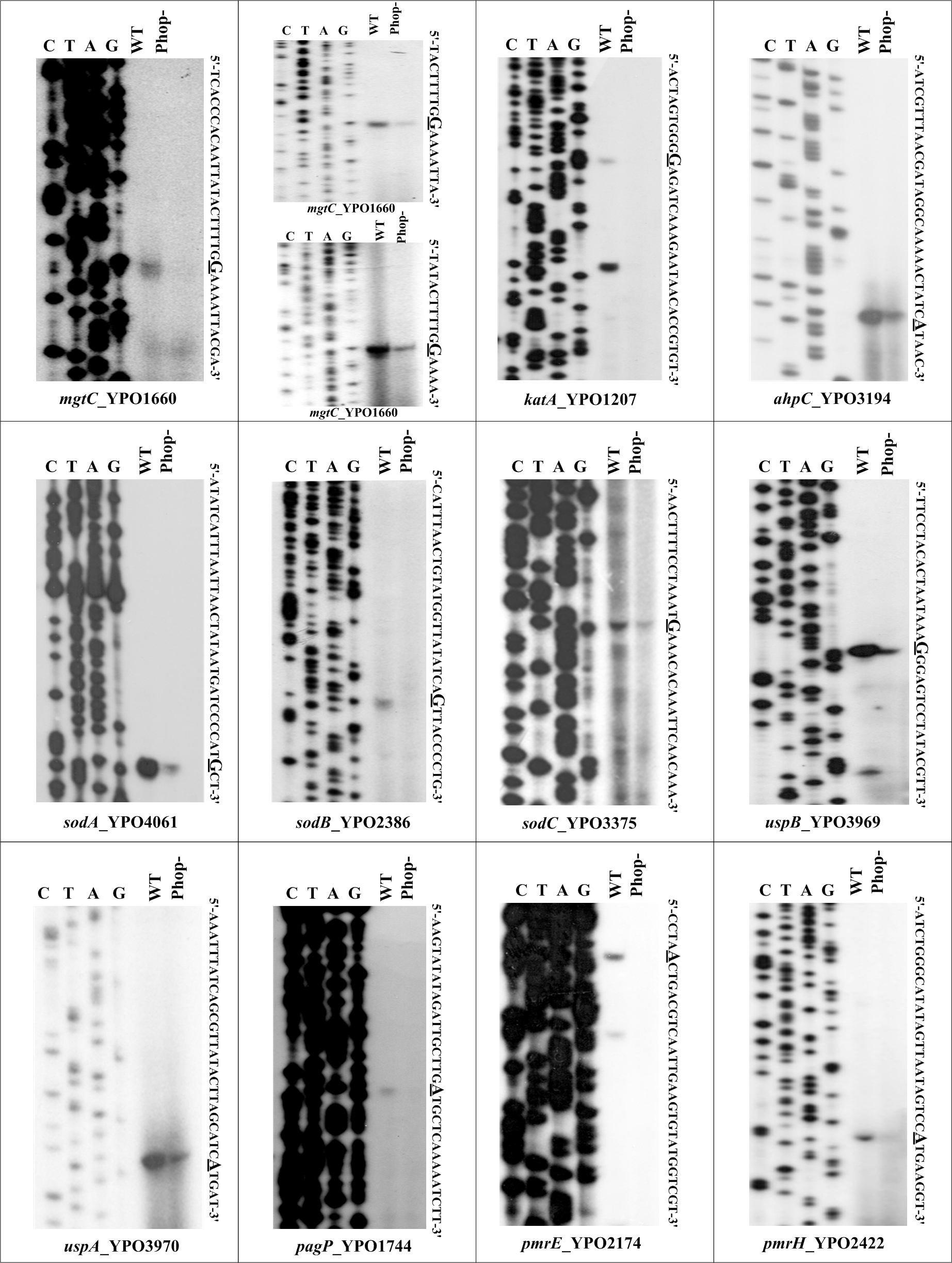

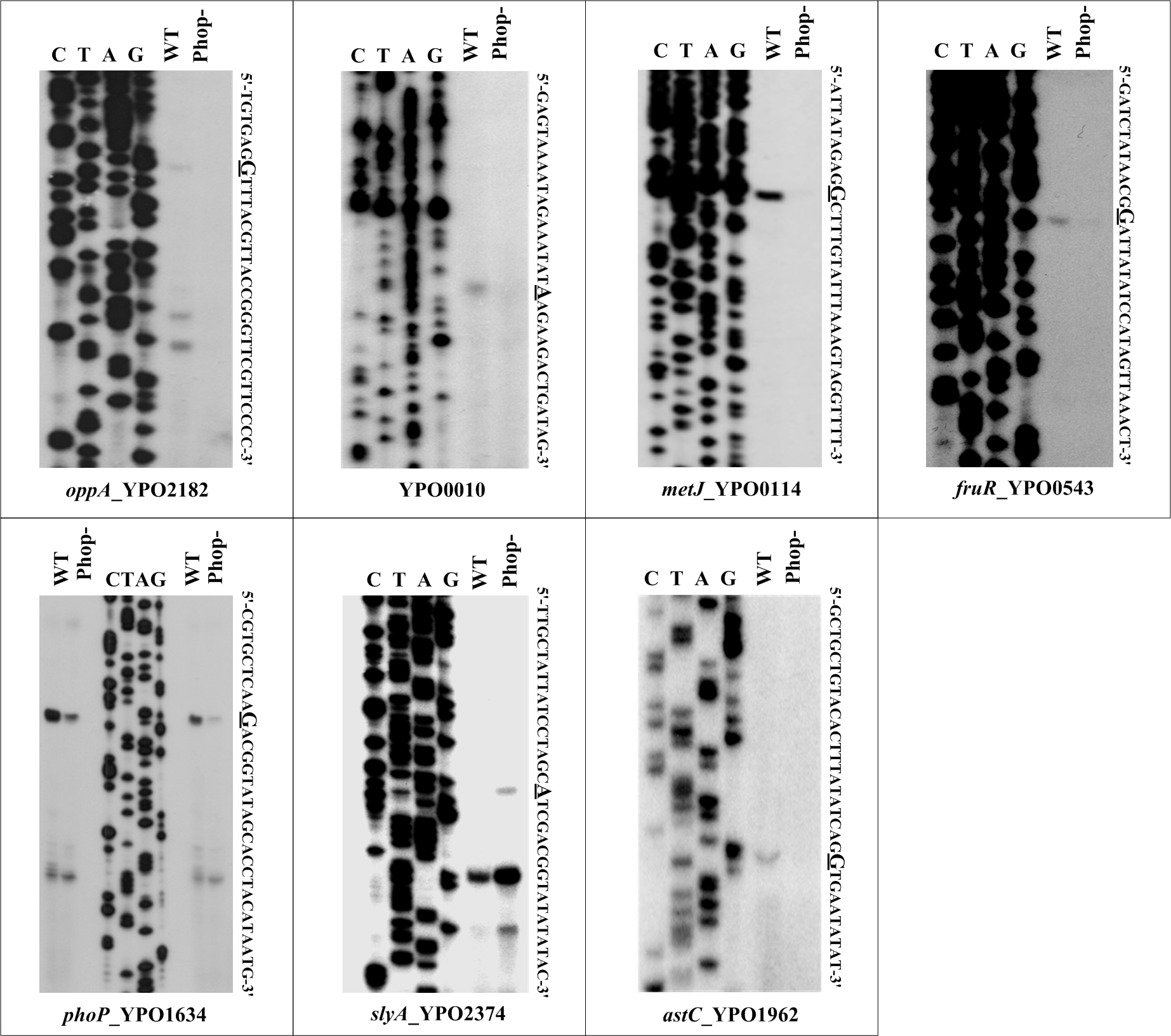

Supplement: Additional file 4 — Primer extension assays. Primer extension was performed by using RNA isolated from the exponential-phase of both the wild-type (WT) and phoP null mutant (phoP-) of Y. pestis grown in the chemically defined TMH medium with 10 μM of Mg2+. An oligonucleotide primer was designed to be complementary to the RNA transcript of each gene at a suitable position. The primer extension products were analyzed with a 6% acrylamide sequencing gel. Lanes C, T, A and G represented the Sanger sequencing reactions. The yield and length of each primer extension product can be used to map the 5' terminus of the RNA transcript, and thus the transcription start site. The transcription start sites were underlined. [file 1471-2164-9-143-S4.doc]

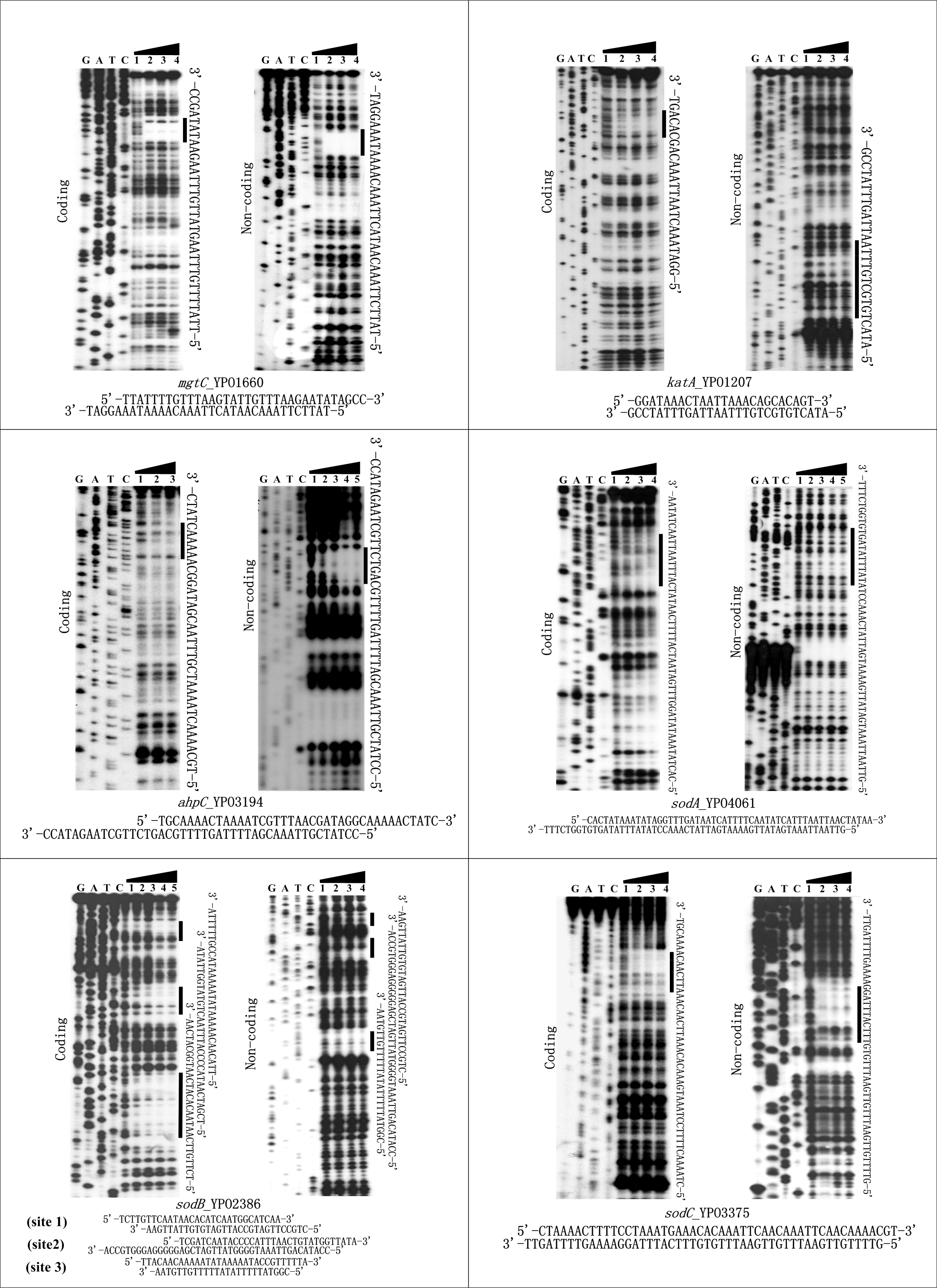

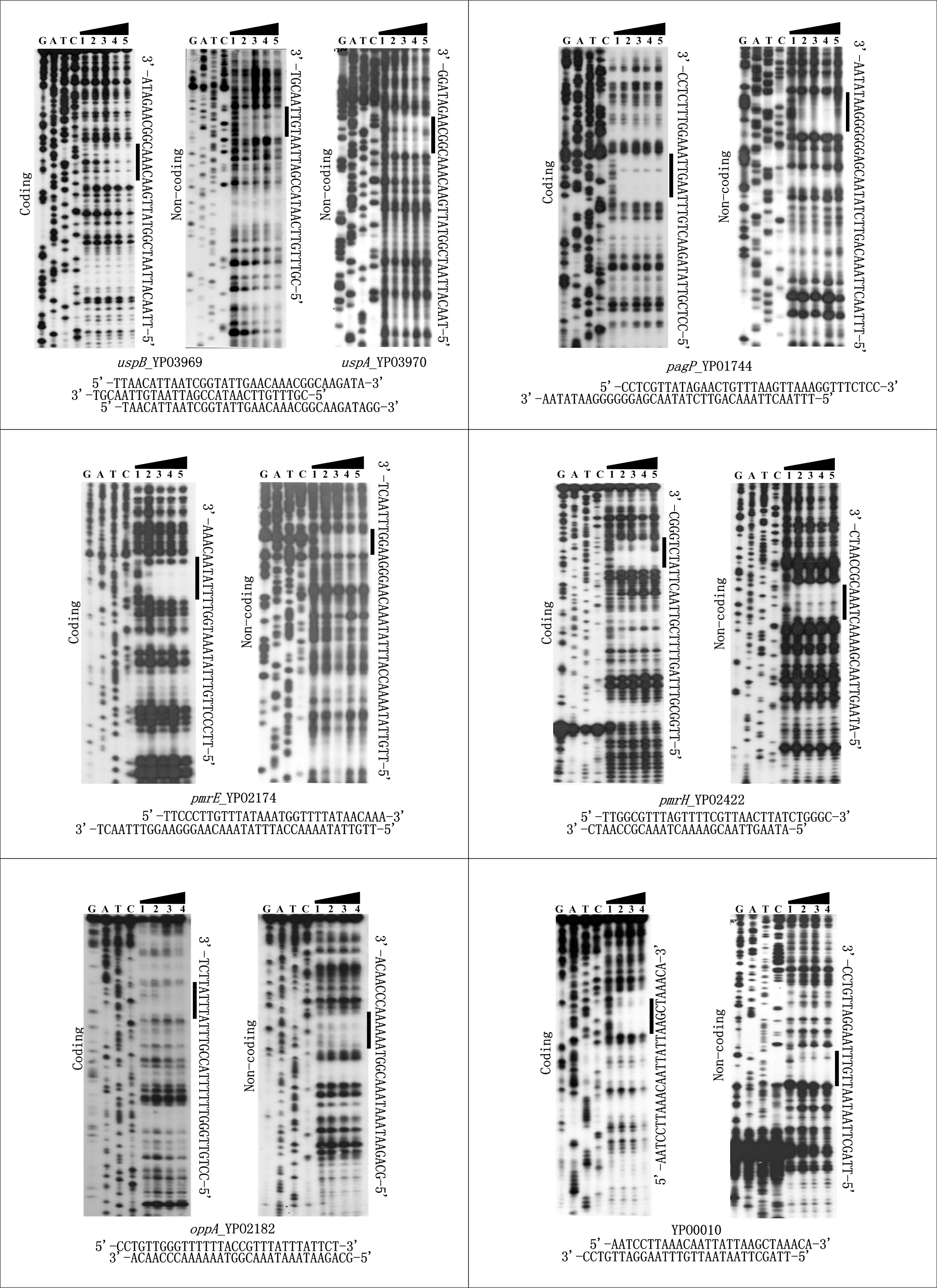

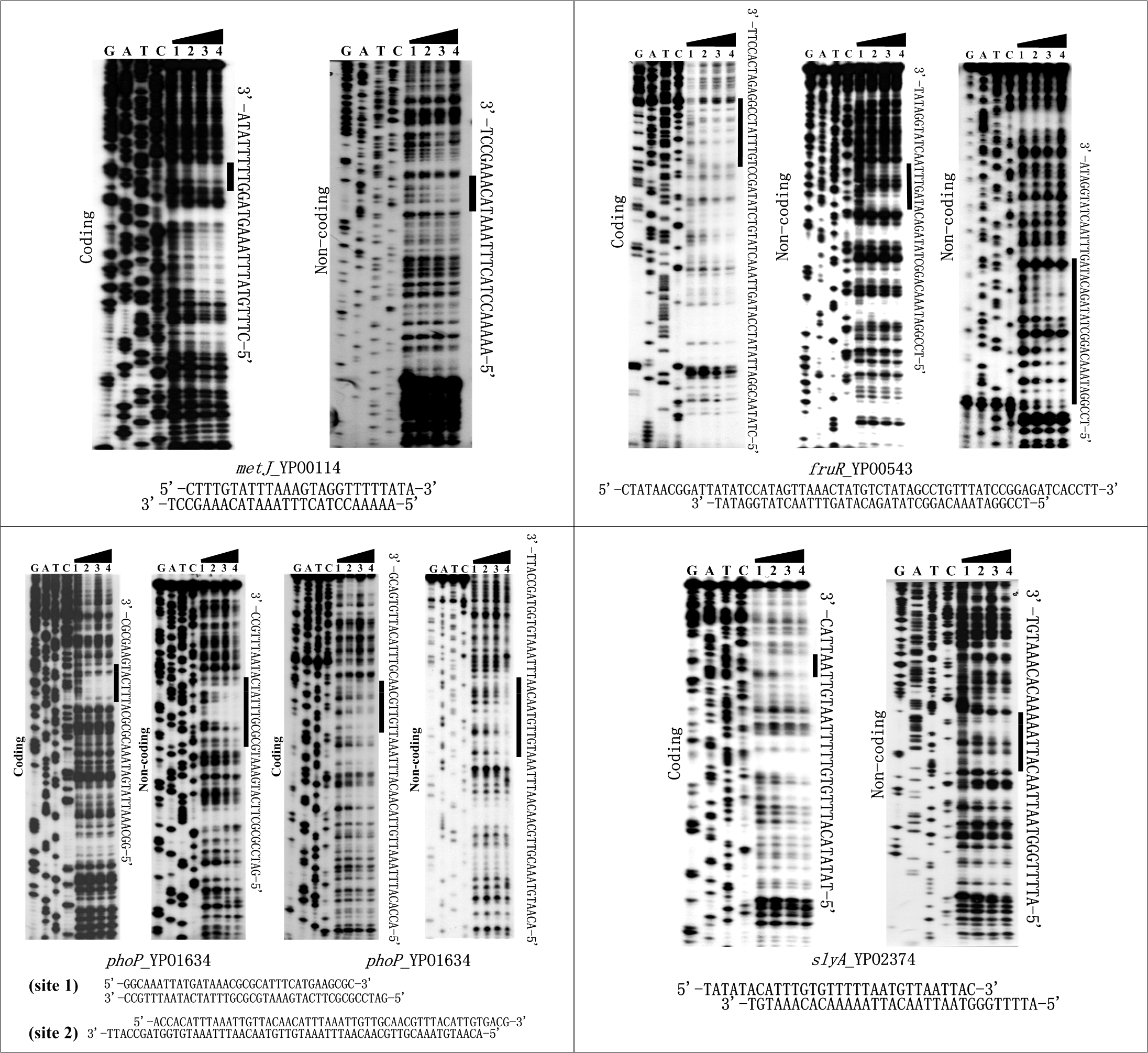

Supplement: Additional file 6 — DNase I footprinting assays. The DNase I footprinting assay was performed on both the coding and noncoding strand of the promoter fragments generated by PCR. The radio-labeled promoter DNA was incubated with increasing amount of the His-PhoP (lanes 1, 2, 3, 4, and 5 contained 0, 5, 15, 25 and 35 pmol, respectively). After the partial digestion with DNase I, the resulting fragments are analyzed by denaturing gel electrophoresis. Lanes G, A, T and C represented the Sanger sequence reactions. On the right-hand side, the PhoP protected regions (bold line) were indicated, and the corresponding DNA sequences of footprints were shown from the bottom (5') to the top (3'). The overlapping of footprints on both strands of each gene was shown at the bottom. [file 1471-2164-9-143-S6.doc]

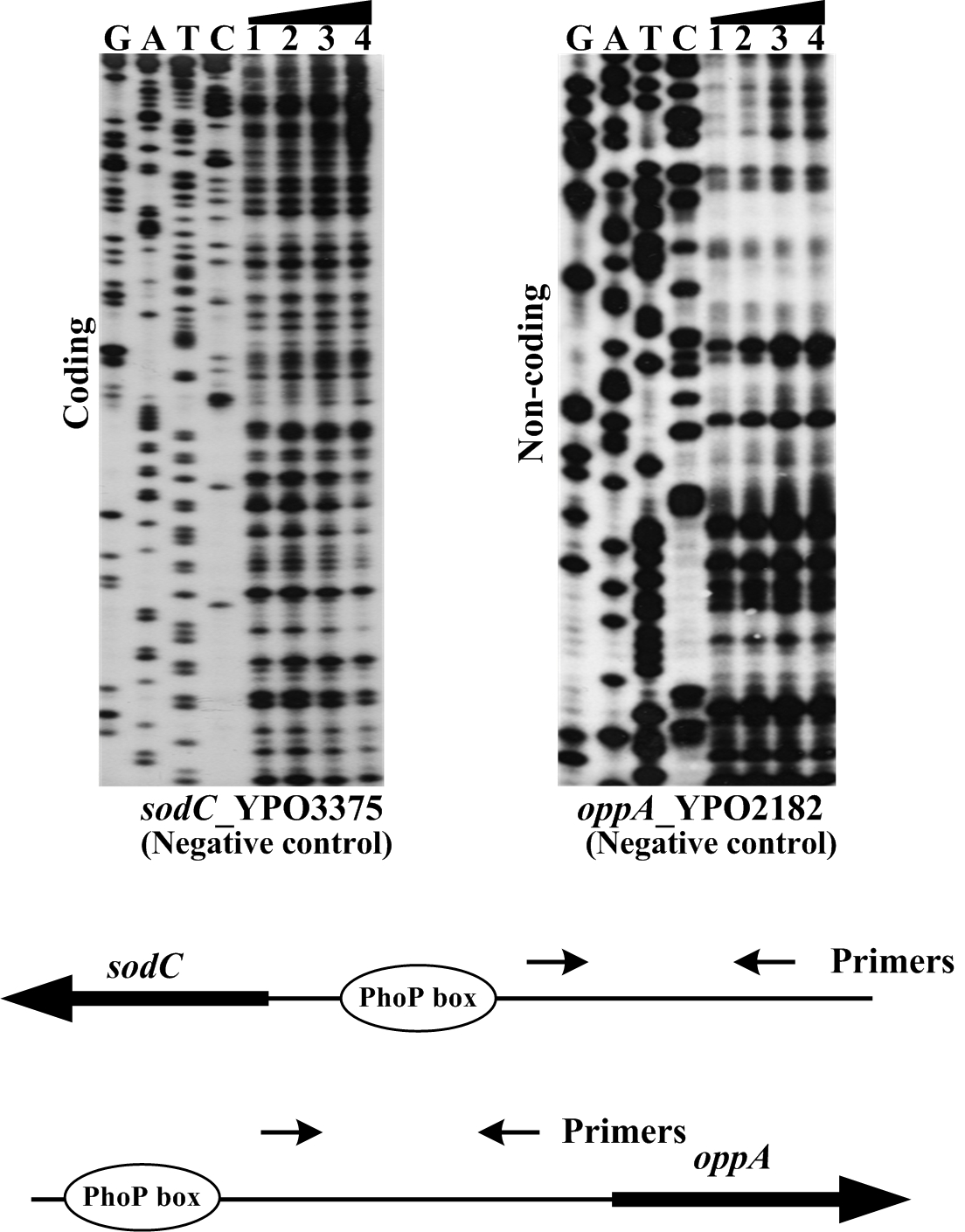

Supplement: Additional file 7 — Negative controls for DNase I footprinting assays. See Additional file 6 for the technical notes. The His tag fused in the recombinant PhoP protein is positively charged, while DNA probe in the footprinting experiments is negatively charged. One may argue that there would be the possibility of specific interaction between His tag and DNA probe. Accordingly, the PCR-generated upstream DNA fragments of two genes sodC and oppA were employed as negative controls. The DNA probes used here did not harbor the predicted PhoP box, compared with the corresponding ones in Additional file 6. No His-PhoP protected footprint region was detected for non-coding or coding strand of sodC and oppA, indicating the specificity of DNase I footprinting experiments. [file 1471-2164-9-143-S7.doc]
