# Supplementary material for: Identification and characterization of PhoP regulon members in Yersinia pestis biovar Microtus
Source: BMC Genomics. 2008 Mar 27;9:143. doi: 10.1186/1471-2164-9-143 (PMC2322996; doi:10.1186/1471-2164-9-143)
Supplement: Additional file 5 — Raw data for real-time PCR. [file 1471-2164-9-143-S5.doc]

**Supplementary Table S2. Raw data for r**eal-time PCR

| Gene ID | Gene name | Crossing Point for WT | Concentration for WT | Crossing Point for *∆phoP* | Concentration for *∆phoP* | Fold change | Readjust | Average |
| --- | --- | --- | --- | --- | --- | --- | --- | --- |
| YPO1660 | *mgtC* | 24.73 | 1375.56 | 28.32 | 128.22 | 0.09 | -10.73 | -11.16 |
|  |  | 21.22 | 5641.17 | 25.18 | 545.76 | 0.10 | -10.34 |  |
|  |  | 22.43 | 2416.13 | 26.76 | 194.54 | 0.08 | -12.42 |  |
| YPO1207 | *katA* | 22.14 | 7392.89 | 24.91 | 1238.25 | 0.17 | -5.97 | -6.44 |
|  |  | 21.50 | 4684.09 | 24.91 | 651.34 | 0.14 | -7.19 |  |
|  |  | 22.86 | 1820.70 | 26.11 | 295.40 | 0.16 | -6.16 |  |
| YPO3194 | *ahpC* | 19.00 | 56826.35 | 20.63 | 21474.51 | 0.38 | -2.65 | -2.86 |
|  |  | 19.85 | 14060.00 | 21.97 | 4455.00 | 0.32 | -3.16 |  |
|  |  | 20.18 | 10560.41 | 22.09 | 3789.65 | 0.36 | -2.79 |  |
| YPO4061 | *sodA* | 20.41 | 22812.40 | 24.72 | 1404.59 | 0.06 | -16.24 | -17.53 |
|  |  | 18.33 | 38760.00 | 23.00 | 2266.00 | 0.06 | -17.11 |  |
|  |  | 17.92 | 46510.50 | 22.80 | 2415.35 | 0.05 | -19.26 |  |
| YPO2386 | *sodB* | 28.41 | 125.42 | 32.12 | 10.23 | 0.08 | -12.26 | -14.34 |
|  |  | 27.66 | 78.11 | 32.46 | 4.62 | 0.06 | -16.91 |  |
|  |  | 26.51 | 165.00 | 31.16 | 11.90 | 0.07 | -13.87 |  |
| YPO3375 | *sodC* | 24.77 | 1341.03 | 27.07 | 293.95 | 0.22 | -4.56 | -4.92 |
|  |  | 24.29 | 731.29 | 27.32 | 134.05 | 0.18 | -5.46 |  |
|  |  | 23.91 | 910.22 | 26.78 | 192.35 | 0.21 | -4.73 |  |
| YPO3969 | *uspB* | 23.30 | 3484.47 | 25.46 | 858.23 | 0.25 | -4.06 | -3.72 |
|  |  | 22.96 | 1777.37 | 25.26 | 515.37 | 0.29 | -3.45 |  |
|  |  | 24.13 | 2023.27 | 25.12 | 552.05 | 0.27 | -3.67 |  |
| YPO3970 | *uspA* | 23.82 | 2472.58 | 26.32 | 484.25 | 0.20 | -5.11 | -4.16 |
|  |  | 21.84 | 3747.16 | 24.03 | 1157.50 | 0.31 | -3.24 |  |
|  |  | 22.16 | 2870.63 | 24.76 | 694.25 | 0.24 | -4.13 |  |
| YPO2174 | *ugd/pmrE* | 26.27 | 503.15 | 28.41 | 120.37 | 0.24 | -4.18 | -4.15 |
|  |  | 25.44 | 341.09 | 27.95 | 88.77 | 0.26 | -3.84 |  |
|  |  | 24.53 | 605.21 | 27.32 | 136.57 | 0.23 | -4.43 |  |
| YPO2422 | *pmrH* | 27.65 | 205.79 | 32.05 | 10.71 | 0.05 | -19.22 | -25.00 |
|  |  | 27.54 | 84.21 | 33.05 | 3.14 | 0.04 | -26.81 |  |
|  |  | 26.99 | 121.04 | 32.81 | 4.18 | 0.03 | -28.96 |  |
| YPO2182 | *oppA* | 23.09 | 3986.52 | 26.17 | 534.77 | 0.13 | -7.45 | -7.00 |
|  |  | 23.61 | 1151.80 | 26.89 | 177.55 | 0.15 | -6.49 |  |
|  |  | 22.21 | 2789.47 | 25.65 | 394.98 | 0.14 | -7.06 |  |
| YPO1715 | *ybjR* | 24.30 | 1817.88 | 26.91 | 327.05 | 0.18 | -5.56 | -5.13 |
|  |  | 23.21 | 1500.98 | 26.11 | 295.64 | 0.20 | -5.08 |  |
|  |  | 22.36 | 2520.46 | 25.19 | 530.34 | 0.21 | -4.75 |  |
| YPO0010 |  | 24.53 | 1560.28 | 26.80 | 353.10 | 0.23 | -4.42 | -4.37 |
|  |  | 25.07 | 437.05 | 27.65 | 107.86 | 0.25 | -4.05 |  |
|  |  | 21.70 | 3886.51 | 24.46 | 838.71 | 0.22 | -4.63 |  |
| YPO0114 | *metJ* | 24.44 | 1659.99 | 28.24 | 135.17 | 0.08 | -12.28 | -11.83 |
|  |  | 24.01 | 881.65 | 28.03 | 84.41 | 0.10 | -10.44 |  |
|  |  | 25.49 | 323.25 | 29.98 | 25.30 | 0.08 | -12.78 |  |
| YPO0414 |  | 27.12 | 289.68 | 29.50 | 58.43 | 0.20 | -4.96 | -5.35 |
|  |  | 27.75 | 73.32 | 31.09 | 11.35 | 0.15 | -6.46 |  |
|  |  | 25.59 | 303.28 | 28.48 | 65.29 | 0.22 | -4.65 |  |
| YPO0543 | *fruR* | 24.26 | 1867.64 | 26.13 | 552.05 | 0.30 | -3.38 | -3.14 |
|  |  | 24.27 | 745.54 | 26.44 | 239.17 | 0.32 | -3.12 |  |
|  |  | 23.12 | 1533.40 | 25.20 | 524.30 | 0.34 | -2.92 |  |
| YPO0736 |  | 27.89 | 175.45 | 29.22 | 70.58 | 0.40 | -2.49 | -3.13 |
|  |  | 30.52 | 31.83 | 31.63 | 7.97 | 0.25 | -3.99 |  |
|  |  | 26.48 | 168.66 | 28.67 | 58.09 | 0.34 | -2.90 |  |
| YPO0849 | *lacI* | 24.55 | 1538.11 | 26.25 | 507.09 | 0.33 | -3.03 | -2.62 |
|  |  | 23.26 | 1459.29 | 24.88 | 662.59 | 0.45 | -2.20 |  |
|  |  | 24.00 | 858.40 | 25.95 | 326.71 | 0.38 | -2.63 |  |
| YPO1279 |  | 23.24 | 3614.71 | 25.94 | 622.63 | 0.17 | -5.81 | -6.27 |
|  |  | 22.45 | 2499.00 | 25.93 | 334.20 | 0.13 | -7.48 |  |
|  |  | 22.01 | 3170.41 | 25.06 | 573.36 | 0.18 | -5.53 |  |
| YPO1634 | *phoP* | 26.29 | 497.51 | 34.96 | 1.54 | 0.00 | -324.01 | -292.58 |
|  |  | 24.08 | 843.88 | 33.14 | 2.96 | 0.00 | -285.29 |  |
|  |  | 25.14 | 405.25 | 34.41 | 1.51 | 0.00 | -268.45 |  |
| YPO2374 | *slyA* | 21.54 | 10905.42 | 18.96 | 64884.70 | 5.95 | 5.95 | 5.51 |
|  |  | 21.65 | 4244.90 | 19.93 | 16990.00 | 4.00 | 4.00 |  |
|  |  | 20.84 | 6831.46 | 18.20 | 44962.83 | 6.58 | 6.58 |  |
| YPO0017 | *polA* | 22.73 | 5043.90 | 25.04 | 1140.00 | 0.23 | -4.42 | -4.15 |
|  |  | 22.49 | 2422.99 | 24.96 | 629.80 | 0.26 | -3.85 |  |
|  |  | 23.00 | 1652.10 | 25.65 | 395.55 | 0.24 | -4.18 |  |
| YPO0498 |  | 27.63 | 207.76 | 31.57 | 14.77 | 0.07 | -14.06 | -15.18 |
|  |  | 25.25 | 386.22 | 29.86 | 25.38 | 0.07 | -15.22 |  |
|  |  | 25.13 | 410.29 | 29.98 | 25.21 | 0.06 | -16.27 |  |
| YPO0860 |  | 29.20 | 74.94 | 30.21 | 36.32 | 0.48 | -2.06 | -2.23 |
|  |  | 29.04 | 31.11 | 30.66 | 15.01 | 0.48 | -2.07 |  |
|  |  | 28.28 | 51.61 | 30.33 | 20.15 | 0.39 | -2.56 |  |
| YPO1937 | *ansP* | 26.61 | 403.48 | 23.59 | 2989.47 | 7.41 | 7.41 | 7.20 |
|  |  | 26.22 | 202.61 | 23.43 | 1712.80 | 8.45 | 8.45 |  |
|  |  | 24.39 | 667.00 | 22.08 | 3823.00 | 5.73 | 5.73 |  |
| YPO1962 | *astC* | 29.36 | 67.71 | 32.47 | 8.09 | 0.12 | -8.36 | -9.03 |
|  |  | 29.99 | 16.61 | 34.01 | 1.68 | 0.10 | -9.90 |  |
|  |  | 29.22 | 27.86 | 33.25 | 3.16 | 0.11 | -8.81 |  |
| YPO2168 | *xthA* | 29.77 | 51.67 | 32.08 | 10.48 | 0.20 | -4.93 | -5.32 |
|  |  | 28.89 | 34.52 | 32.16 | 5.63 | 0.16 | -6.13 |  |
|  |  | 27.76 | 72.65 | 30.81 | 14.88 | 0.20 | -4.88 |  |
| YPO3766 | *fadB* | 21.65 | 10192.51 | 23.35 | 3492.77 | 0.34 | -2.92 | -3.15 |
|  |  | 19.50 | 17700.00 | 21.71 | 5280.00 | 0.30 | -3.35 |  |
|  |  | 19.08 | 21704.31 | 21.16 | 6832.21 | 0.31 | -3.18 |  |
| YPO4116 | *pstC* | 23.46 | 3139.81 | 26.20 | 524.30 | 0.17 | -5.99 | -6.46 |
|  |  | 21.31 | 5317.60 | 24.51 | 846.50 | 0.16 | -6.28 |  |
|  |  | 22.45 | 2378.95 | 25.91 | 334.20 | 0.14 | -7.12 |  |
