# Supplementary material for: Identification and characterization of PhoP regulon members in Yersinia pestis biovar Microtus
Source: BMC Genomics. 2008 Mar 27;9:143. doi: 10.1186/1471-2164-9-143 (PMC2322996; doi:10.1186/1471-2164-9-143)
Supplement: Additional file 8 — Direct PhoP targets in Escherichia coli and Salmonella typhimurium. [file 1471-2164-9-143-S8.doc]

***Supplementary Table S3. Direct PhoP targets in Escherichia coli and*** Salmonella typhimurium

| **Gene** | **Product** | ***E. coli*** | ***S. typhimurium*** | **Reference** |
| --- | --- | --- | --- | --- |
| *hdeA* | Acid-resistance protein | + |  | [1] |
| *hemL* | Glutamate-1-semialdehyde 2,1-aminomutase | + |  | [2] |
| *macAB* | Drug efflux system |  | + | [3] |
| *mgrB* | Low magnesium induced hypothetical protein | + |  | [2] |
| *mig-14* | Inner membrane-associated protein, required for antimicrobial peptide resistance |  | + | [1] |
| *mgtA* | Mg^2+^-importing ATPase | + | + | [2,4,5] |
| *mgtCB* | Mg^2+^-importing ATPase | + | + | [1,2] |
| *nagA* | N-acetylglucosamine-6-phosphate deacetylase | + |  | [2] |
| *pagC* | Outer membrane protein |  | + | [1] |
| *pcgL* | D-alanyl-D-alanine dipeptidase |  | + | [5] |
| *phoPQ* | Two-component virulence regulatory system | + | + | [2,5] |
| *pmrD* | Polymyxin B resistance protein |  | + | [5] |
| *rstAB* | Two-component regulatory system | + |  | [2] |
| *slyA* | Virulence-related transcriptional regulator |  | + | [6] |
| *slyB*/*pcgH* | Outer membrane lipoprotein | + | + | [2,5] |
| *somA* | *Salmonella* virulence determinant |  | + | [3] |
| *ssrB*-s*piR* | Two-component virulence regulatory system |  | + | [7] |
| *treR* | Trehalose operon transcriptional repressor | + |  | [4] |
| *ugtL* | Inner membrane protein, required for antimicrobial peptide resistance |  | + | [6] |
| *vboR* | Hypothetical protein | + |  | [2] |
| *yhiW*/g*adW* | AraC-type regulatory protein, required for acid resistance | + |  | [1] |
| *yrbL* | Putative cytoplasmic protein | + |  | [2] |

‘+’ represents the detection of the gene in the corresponding bacteria, by gel mobility shift assay and/or DNase I footprinting

**Reference**

1. Zwir I, Shin D, Kato A, Nishino K, Latifi T, et al. (2005) Dissecting the PhoP regulatory network of Escherichia coli and Salmonella enterica. Proc Natl Acad Sci U S A 102: 2862-2867.

2. Minagawa S, Ogasawara H, Kato A, Yamamoto K, Eguchi Y, et al. (2003) Identification and molecular characterization of the Mg2+ stimulon of Escherichia coli. J Bacteriol 185: 3696-3702.

3. Nishino K, Latifi T, Groisman EA (2006) Virulence and drug resistance roles of multidrug efflux systems of Salmonella enterica serovar Typhimurium. Mol Microbiol 59: 126-141.

4. Yamamoto K, Ogasawara H, Fujita N, Utsumi R, Ishihama A (2002) Novel mode of transcription regulation of divergently overlapping promoters by PhoP, the regulator of two-component system sensing external magnesium availability. Mol Microbiol 45: 423-438.

5. Lejona S, Aguirre A, Cabeza ML, Garcia Vescovi E, Soncini FC (2003) Molecular characterization of the Mg2+-responsive PhoP-PhoQ regulon in Salmonella enterica. J Bacteriol 185: 6287-6294.

6. Shi Y, Latifi T, Cromie MJ, Groisman EA (2004) Transcriptional control of the antimicrobial peptide resistance ugtL gene by the Salmonella PhoP and SlyA regulatory proteins. J Biol Chem 279: 38618-38625.

7. Bijlsma JJ, Groisman EA (2005) The PhoP/PhoQ system controls the intramacrophage type three secretion system of Salmonella enterica. Mol Microbiol 57: 85-96.
