# Supplementary material for: Identification and characterization of PhoP regulon members in Yersinia pestis biovar Microtus
Source: BMC Genomics. 2008 Mar 27;9:143. doi: 10.1186/1471-2164-9-143 (PMC2322996; doi:10.1186/1471-2164-9-143)
Supplement: Additional file 9 — Distribution of PhoP-dependent genes in Yersinia pestis as revealed by microarray. [file 1471-2164-9-143-S9.doc]

**Supplementary Table S4. Distribution of PhoP-dependent genes in *Yersinia pestis*** as revealed by microarray

| **Function class** | **Number of genes** | |
| --- | --- | --- |
|  | **PhoP-activated** | **PhoP-repressed** |
| **Small molecule metabolism** | 132 | 27 |
| Degradation of small molecules | 18 | 3 |
| Energy metabolism | 53 | 5 |
| Central intermediary metabolism | 13 | 3 |
| Amino acid biosynthesis | 17 | 4 |
| Polyamine synthesis | 0 | 1 |
| Purines, pyrimidines, nucleosides and nucleotides | 12 | 5 |
| Biosynthesis of cofactors, prosthetic groups and carriers | 13 | 4 |
| Fatty acid biosynthesis | 6 | 2 |
| **Macromolecule metabolism** | 84 | 127 |
| Synthesis and modification of macromolecules | 19 | 71 |
| Degradation of macromolecules | 16 | 6 |
| Cell envelop | 49 | 50 |
| **Broad regulatory function** | 17 | 9 |
| **Cell process** | 56 | 53 |
| Transport/binding proteins | 40 | 41 |
| Chaperones, chaperonins, heat shock | 5 | 1 |
| Cell division | 0 | 5 |
| Chemotaxis and mobility | 0 | 4 |
| Detoxification | 6 | 0 |
| Pathogenicity | 5 | 2 |
| **Others** | 114 | 87 |
| Phage-related functions and prophage | 1 | 4 |
| Drug/analogue sensitivity | 3 | 2 |
| Adaptation and atypical conditions | 13 | 5 |
| Unknown | 80 | 55 |
| Plasmid pCD1 | 10 | 8 |
| Plasmid pMT1 | 4 | 6 |
| Plasmid pPCP1 | 2 | 2 |
| Plasmid pCRY | 1 | 5 |
