# Supplementary material for: Identification and characterization of PhoP regulon members in Yersinia pestis biovar Microtus
Source: BMC Genomics. 2008 Mar 27;9:143. doi: 10.1186/1471-2164-9-143 (PMC2322996; doi:10.1186/1471-2164-9-143)
Supplement: Additional file 10 — Predicted direct PhoP target genes in Yersinia pestis by matrix matching. [file 1471-2164-9-143-S10.doc]

**Supplementary Table S5. Predicted direct PhoP target genes in *Yersinia pestis* by matrix matching**

| **score** | **Microarray** | | **Gene ID** | **Gene name** | **Product** |
| --- | --- | --- | --- | --- | --- |
| ***Synthesis and modification of macromolecules*** | | | | | |
| 8.13 | 3.02 | | YPO0111 | *rpmE* | 50S ribosomal protein L31 |
|  | 3.35 | | | YPO0643 | | --- | | *rpoD* | RNA polymerase sigma factor RpoD |
|  | 8.15 | | YPO0644 | *dnaG* | DNA primase |
| 7.52 | 3.13 | | YPO0645 | *rpsU* | 30S ribosomal protein S21 |
| 7.14 | 4.58 | | | YPO1044 | | --- | | *rpsB* | 30S ribosomal protein S2 |
|  | 3.42 | | YPO1045 | *tsf* | elongation factor Ts |
|  | 3.26 | | YPO1046 | *pyrH* | uridylate kinase |
|  | 2.35 | | YPO1047 | *frr* | ribosome recycling factor |
| 7.17 | 3.45 | | YPO1392 | *rpsA* | 30S ribosomal protein S1 |
| 7.34 | -2.31 | | YPO2610 | *leuS* | leucyl-tRNA synthetase |
| 7 | -2.89 | | | 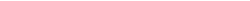YPO3354 | | --- | | *mutS* | DNA mismatch repair protein MutS |
| ***Amino acid biosynthesis*** | | | | | |
| 8 | -5.80 | | YPO0287 |  | putative methylenetetrahydrofolate reductase |
|  | -5.84 | | | YPO1547 | | --- | | *hisC* | histidinol-phosphate aminotransferase |
| 9.6 | -3.31 | | YPO1548 | *hisD* | histidinol dehydrogenase |
| 7.89 | 3.61 | | YPO1687 |  | putative alanine racemase |
| ***Degradation of small molecule and macromolecules*** | | | | | |
| 9.09 | -6.12 | | YPO0253 | *acs* | acetyl-coenzyme A synthetase |
| 7.17 | -2.20 | | | YPO1761 | | --- | | *hpaG* | 4-hydroxyphenylacetate degradation bifunctional isomerase/decarboxylase (pseudogene) |
|  | -2.63 | | YPO1763 | *hpaE* | 5-carboxymethyl-2-hydroxymuconate semialdehyde dehydrogenase |
| 7.6 | -4.11 | | YPO1972 | *hutI* | imidazolonepropionase |
| 7.67 | 2.81 | | YPO1231 | *pla2* | putative outer membrane-associated protease |
| 7.25 | 2.85 | | YPO2946 | *clpB5* | Clp ATPase |
| 7.14 | 2.34 | | YPO3275 | *clpB* | Clp ATPase |
| 7.1 | -3.97 | | YPO3975 | *opdA* | oligopeptidase A |
| ***Cell envelop*** | | | | | |
| 9.09 | 2.20 | | YPO0254 | *gltP* | proton glutamate symport protein |
| 7.32 | 3.75 | | YPO0902 |  | putative surface protein (partial) |
| 7.55 | -2.41 | | YPO0987 |  | putative exported protein |
| 7.38 | 3.72 | | | YPO1420 | | --- | | *pqiA* | putative paraquat-inducible protein A |
|  | 2.99 | | YPO1421 | *pqiB* | putative paraquat-inducible protein B |
| 7.29 | -10.98 | | YPO1635 |  | putative lipoprotein |
| 8.07 | 2.73 | | YPO1686 |  | putative exported protein |
| 7.01 | 3.73 | | YPO2190 | *ail* | attachment invasion locus protein precursor |
| 7.77 | -5.83 | | YPO2292 |  | putative lipoprotein |
| 7.04 | 2.62 | | YPO2922 |  | putative membrane protein |
| 8.28 | 4.16 | | YPO3021 |  | putative outer membrane protein (pseudogene) |
| 8.12 | -3.09 | | YPO3050 |  | putative exported protein |
| 8.13 | -4.74 | | YPO3111 | *wbyH* | putative exported protein |
| ***Energy metabolism*** | | | | | |
| 7.31 | -7.58 | | YPO1108 | *gltA* | citrate synthase GltA |
| 7.31 | -4.02 | | | YPO1109 | | --- | | *sdhC* | succinate dehydrogenase cytochrome b-556 subunit |
|  | -4.19 | | YPO1110 | *sdhD* | succinate dehydrogenase hydrophobic membrane anchor protein |
|  | -13.20 | | YPO1111 | *sdhA* | succinate dehydrogenase flavoprotein subunit |
|  | -8.98 | | YPO1112 | *sdhB* | succinate dehydrogenase iron-sulfur protein |
|  |  | |  |  |  |
|  | -27.37 | | YPO1113 | *sucA* | 2-oxoglutarate dehydrogenase E1 component |
|  | -8.22 | | YPO1114 | *sucB* | dihydrolipoamide succinyltransferase component of 2-oxoglutarate dehydrogenase complex |
|  | -9.46 | | YPO1115 | *sucC* | succinyl-CoA synthetase beta chain |
|  | -3.61 | | YPO1116 | *sucD* | succinyl-CoA synthetase alpha chain |
|  | -3.84 | | YPO1117 | *cydA* | cytochrome D ubiquinol oxidase subunit I |
|  | -2.98 | | YPO1118 | *cydB* | cytochrome D ubiquinol oxidase subunit II |
| 9.64 | -2.35 | | YPO1177 | *dld* | D-lactate dehydrogenase |
| 8.36 | -3.15 | | YPO1541 | *gnd* | 6-phosphogluconate dehydrogenase, decarboxylating |
| 7 | -3.50 | | YPO2066 | *zwf* | glucose-6-phosphate 1-dehydrogenase |
| 7.92 | -3.28 | | YPO2157 | *gapA* | glyceraldehyde 3-phosphate dehydrogenase A |
| 7.98 | -5.39 | | YPO2180 | *adhE* | aldehyde-alcohol dehydrogenase |
| 7.26 | -2.87 | | YPO2264 | *fumC* | fumarate hydratase, class II |
|  | -3.40 | | | YPO2543 | | --- | | *nuoN* | NADH dehydrogenase I chain N |
|  | -3.29 | | YPO2544 | *nuoM* | NADH dehydrogenase I chain M |
|  | -7.26 | | YPO2545 | *nuoL* | NADH dehydrogenase I chain L |
|  | -8.93 | | YPO2547 | *nuoJ* | NADH dehydrogenase I chain J |
|  | -6.57 | | YPO2548 | *nuoI* | NADH Dehydrogenase I chain I |
|  | -3.84 | | YPO2549 | *nuoH* | NADH dehydrogenase I chain H |
|  | -4.75 | | YPO2550 | *nuoG* | NADH dehydrogenase I chain G |
|  | -3.83 | | YPO2551 | *nuoF* | NADH dehydrogenase I chain F |
|  | -3.07 | | YPO2552 | *nuoE* | NADH dehydrogenase I chain E |
| 7.14 | -2.29 | | YPO2553 | *nuoD* | NADH dehydrogenase I chain C/D |
| 7.51 | -2.80 | | YPO3168 | *cyoE* | protoheme IX farnesyltransferase |
| 7.29 | -4.38 | | YPO3321 | *cybB* | probable cytochrome B561 |
| ***Transport/binding proteins*** | | | | | |
| 7.96 | 2.75 | | YPO0798 |  | putative sugar transport protein |
| 7.39 | 3.43 | | YPO1004 | *yapH* | putative autotransporter protein |
| 8.39 | 3.19 | | YPO2392 |  | putative transport protein |
| 7.2 | 2.06 | | YPO2615 | *glnH* | putative amino acid-binding protein precursor |
| 7.75 | 6.58 | | YPO2774 | *hisJ* | histidine-binding periplasmic protein |
| 7.53 | -3.32 | | YPO2846 |  | putitive ABC-transporter ATP-binding protein |
| 8.73 | 5.85 | | YPO2886 | *yapA* | putative autotransporter protein |
| 7.46 | 5.23 | | YPO2904 | *hcaT* | putative transport permease protein |
|  | -5.88 | | | YPO3010 | | --- | |  | hypothetical protein |
|  | -4.92 | | YPO3012 | *cysA* | sulfate transport ATP-binding protein |
|  | -2.63 | | YPO3013 | *cysW* | sulfate transport system permease protein CysW |
|  | -4.32 | | YPO3014 | *cysT* | sulfate transport system permease protein CysT |
| 7.01 | -4.44 | | YPO3015 | *cysP* | thiosulfate-binding protein |
| 8.96 | -2.15 | | YPO3633 |  | putative periplasmic binding protein |
| 7.66 | -2.32 | | YPO4003 | *dppA* | periplasmic dipeptide transport protein |
| 7.13 | -2.27 | | YPO4111 |  | putative periplasmic solute-binding protein |
|  |  | |  |  |  |
| ***Broad regulatory functions*** | | | | | |
| 9.4 | 2.05 | | YPO2378 |  | TetR-family transcriptional regulatory protein |
| 7.55 | -2.85 | | YPO3063 | *gcvR* | glycine cleavage system transcriptional repressor |
| ***Various unknown functions*** | | | | | |
| 7.76 | | 2.39 | YPO0307 |  | hypothetical protein |
| 7.96 | | -4.49 | YPO0623 |  | putative aminotransferase |
| 9.54 | | -5.10 | YPO0678 |  | putative iron-containing alcohol dehydrogenase |
| 8.84 | | 3.12 | | 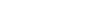YPO0934 | | --- | |  | conserved hypothetical protein |
|  | | -2.77 | YPO0935 | *gshB* | glutathione synthetase |
|  | | -2.96 | YPO0937 |  | conserved hypothetical protein |
|  | | -2.65 | YPO0938 |  | putative carbon-nitrogen hydrolase |
|  | | -2.80 | YPO0939 |  | conserved hypothetical protein |
| 7.08 | | -3.66 | YPO1502 |  | probable alcohol dehydrogenase |
| 7.23 | | -4.88 | YPO1653 |  | probable Zinc-binding dehydrogenase |
| 7.4 | | -2.29 | YPO1991 |  | putative sugar-phosphate isomerase |
| 7.32 | | -18.17 | YPO1994 |  | hypothetical protein |
| 7.09 | | 4.11 | YPO2295 |  | haloacid dehalogenase-like hydrolase family protein |
| 8.05 | | -3.35 | YPO2854 |  | conserved hypothetical protein |
| 8.28 | | 2.53 | YPO3020 |  | putative sugar kinase |
| 7.01 | | 5.76 | YPO3509 |  | putative GTP-binding protein |
| 7.1 | | -2.41 | YPO3976 |  | conserved hypothetical protein |
| 7.15 | | 3.39 | YPO4018 | *cysM* | pyridoxal-phosphate dependent protein |
| 8.12 | | -4.00 | YPO4064 |  | hypothetical protein |
| 7.57 | | 4.52 | YPCD1.17c | *ylpA* | putative lipoprotein precursor pseudogene, ylpA |
| 7 | | 2.02 | YPCD1.59 | *yscJ* | putative type III secretion lipoprotein, yscJ, ylpB |
| 7.78 | | 2.61 | YPCD1.60 | *yscK* | putative type III secretion protein, yscK |
| 7.09 | | -3.52 | YPMT1.34A |  | hypothetical protein |
| 7.33 | | 7.22 | YPMT1.68A |  | hypothetical protein |
| 7.33 | | 8.31 | YPMT1.69 |  | hypothetical protein |
|  | |  |  |  |  |
| ***others*** | |  |  |  |  |
| 7.41 | | 2.98 | YPO0947 |  | putative virulence determinant |
| 7.68 | | -22.49 | YPO3319 | *katY* | catalase-peroxidase |
| 8.29 | | -2.74 | YPO1783 | *ftnA* | ferritin |
| 7.5 | | -2.78 | YPO2323 | *acpD* | acyl carrier protein phosphodiesterase |
| 7.78 | | 2.71 | YPO3411 | *speE* | spermidine synthase |
| 7.07 | | -2.71 | YPO1706 | *htpX* | putative heat shock protein |
| 8.66 | | -2.59 | YPO4084 | *ibpB* | heat shock protein |
